# 9th European Conference on Rare Diseases & Orphan Products (ECRD Vienna 2018): Vienna, Austria. 10-12 May 2018

**DOI:** 10.1186/s13023-018-0895-2

**Published:** 2018-09-28

## Poster presentations

### P1 Rare Diseases in Spain: a nationwide registry-based mortality study

#### Verónica Alonso-Ferreira^1^, Ana Villaverde-Hueso^1^, Greta Arias-Merino^2^, Germán Sánchez-Díaz^2,3^, Manuel Posada de la Paz^1^

##### ^1^Institute of Rare Diseases Research (IIER), Instituto de Salud Carlos III (ISCIII), and Centre for Biomedical Network Research on Rare Diseases (CIBERER), Madrid, Spain; ^2^Institute of Rare Diseases Research (IIER), Instituto de Salud Carlos III (ISCIII), Madrid, Spain; ^3^Department of Geology, Geography and Environmental Sciences, University of Alcala, Alcalá de Henares, Spain

###### **Correspondence:** Verónica Alonso-Ferreira

**Background**

Rare diseases (RD) are still lacking of population-based data and epidemiological indicators. The aim of this study is to assess 15-years’ time trends of mortality attributed to RD in Spain.

Methods

Mortality statistics from the Spanish National Statistics Institute provide population-based data [1]. Deaths due to RD were extracted from official annual databases (1999-2013). Only those ICD-10 codes considered as RD by SpainRDR experts were included in this study [2]. Annual sex- and age-specific adjusted mortality rates per 100,000 inhabitants were calculated and time trends were performed by joinpoint regression analysis.

**Results**

RD mortality represents 1.2% of all registered deaths from 1999 to 2013 in Spain. Mortality attributed to RD is higher in males (51.2%) than females (48.8%). Children (<15 years old) account for 15.2% of deceases. Distribution of RD deaths according to main ICD-10 groups is displayed in Fig. 1.

Regarding time trends of RD mortality (Fig. 2), there has been a 0.95% decline in the annual age-adjusted death rates due to all RD (-0.95%, p<0.001). In addition:

Decrease trends were also observed in the following subgroups: *RD of the blood and blood-forming organs and certain rare disorders involving the immune mechanism* (-2.06%, p<0.001), *RD of the circulatory system* (-3.90%, p<0.01), and *rare congenital malformations, deformations and chromosomal abnormalities* (-5.39%, p<0.01).

Increase trends of annual age-adjusted death rates were detected for *RD of the nervous system* (1.85%, p<0.01), *RD of the respiratory system* (2.39%, p<0.01), *RD of the digestive system* (1.83%, p<0.05) and those *RD affecting the genitourinary system* (9.38% p<0.05).

*Other RD groups* have not showed any significant change in this period.

**Conclusion**

Official mortality statistics share criteria for analysing uniform and robust time series, which is useful for studying low-prevalence diseases. Assessed RD mortality trends are valuable information for the health authorities in Spain.

**Acknowledgements**

Spanish Strategy Action for Health (AESI) supported this research, project No. TPY1238/15. The authors would like to thank the members of the Spanish Rare Diseases Registries Research Network (Spain RDR) for their invaluable expertise in matters pertaining to the International Classification of Diseases and identification of rare diseases.

**References**

1. National Statistics Institute (Instituto Nacional de Estadística), Spanish Statistical Office [http://www.ine.es/en/]

2. Spanish Rare Diseases Registries Research Network (SpainRDR) [https://spainrdr.isciii.es/en/]

**Fig. 1 (abstract P1).**
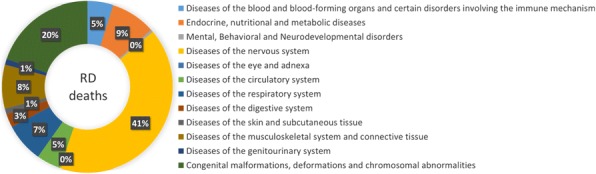
Distribution of RD deaths according to main ICD-10 groups

**Fig. 2 (abstract P1).**
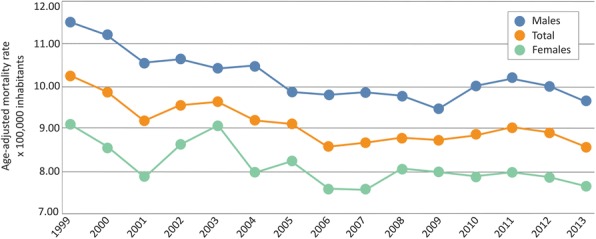
Annual age-adjusted mortality rates attributed to RD by sex

### P2 Share4Rare: Social media platform dedicated to rare diseases, using collective intelligence for the generation of awareness and knowledge

#### Dimitrios Athanasiou^3^, Begonya Nafría^1^, Elizabeth Vroom^2^

##### ^1^Institut de Recerca Sant Joan de Déu and Fundació Sant Joan de Déu, Esplugues de Llobregat, 08950 Spain; ^2^Parent Projects Muscular Dystrophy, Veenendaal, Netherlands; ^3^Parent Projects Muscular Dystrophy, Athens, Greece

**Background**

Share4Rare project is a digital platform dedicated to rare diseases, using collective intelligence for the generation of awareness and advanced knowledge on this large group of diseases.

There are no mandatory patients’ registries of rare disease. There is no real or updated data on the geographical and epidemiological incidence of these diseases. The low number of patients for each disease makes difficult the recruitment, the medical intervention and importantly, the development of new therapeutic options for these patients.

While certain social media are already connecting patients and families around the world, the collection of clinical and psycho-social information is not working conveniently and the real patient empowerment remains elusive.

Share4Rare will be the first social platform to collect trustable resources, information and tools about rare diseases. It will promote the generation of scientific knowledge thanks to clinical data donation from patients and families.

**Methods**

Share4Rare is going to recruit and empower patients and other users linked to rare diseases in two types of communities:

- Social communities. Focused in sharing information, solving doubts for the better care and creating registers of patients. We are going to offer educational tools with the aim to empower in: 1) health care, 2) management of patients’ associations and 3) research.
- Scientific communities. With the aim to offer the opportunity to donate clinical relevant data that can change the course of the disease. This data will make feasible the description of the natural history and create an exhaustive clinical register of patients. Patients and careers are going to work with a methodology of research based on the *collective intelligence* with a three steeps cycle:
  - ° Access to educational chapters about the disease, written with lay language.
  - ° Fill in medical surveys with specific and exhaustive questions regarding the different contents of the medical chapters.
  - ° Statistical reports including the main medical results.

**Results**

Creation of an international community of patients while we erase the geographical, language boundaries.

Digital tools to communicate disseminate and educate in the care and treat of rare diseases.

Increase the social awareness.

Promote other research projects focused on the study of new therapeutic options.

**Conclusions**

The use of social media platforms can change in a positive way the devastating reality of rare diseases. By eliminating the geographical and language boundaries this is a way to avoid the misinformation and isolation that rare disease patients and families face nowadays.

**References**

1. Partnering With Patients in the Development and Lifecycle of Medicines: A Call for Action

2. http://ec.europa.eu/health/files/eudralex/vol-1/reg_2000_141/reg_2000_141_en.pdf

3. http://ec.europa.eu/health/rare_diseases/policy_en

4. 2014 Report on the State of the Art of Rare Disease Activities in Europe: Part I – Overview of Rare Disease Activities in Europe.

5. Aronson J. Rare diseases and orphan drugs Br J Clin Pharmacol. 2006; 61(3): 243-4.

6. COUNCIL RECOMMENDATION of 8 June 2009 on an action in the field of rare diseases (2009/C 151/02).

7. Bavisetty S, et al. Emergence of pediatric rare diseases. Rare Diseases 2013, volume 1.

8. Rare Diseases UK. Key Statistics from the RDUK Report ‘Experiences of Rare Diseases: An Insight from Patients and Families’.

9. Engel PA, et al. Physician and patient perceptions regarding physician training in rare diseases: the need for stronger educational initiatives for physicians. Journal of Rare Disorders 2013: Vol. 1, Issue 2.

10. EURORDIS. The Voice of 12,000 Patients. Experiences and Expectations of Rare Disease Patients on Diagnosis and Care in Europe.

11. Lim L, Nutt S, Sen A. Experiences of Rare Diseases: An Insight from Patients and Families. December 2010.

12. 2014 Report on the State of the Art of Rare Disease Activities in Europe: Part I – Overview of Rare Disease Activities in Europe.

13. COUNCIL RECOMMENDATION of 8 June 2009 on an action in the field of rare diseases (2009/C 151/02).

### P3 Thalassaemia, worldwide

#### Soroya Beacher^1^, Elmaz Citak^1^, Sachith Mettananda^2^, Sanath P Lamabadusuriya^3^, Petra Poulissen^4^, Tessa Risch^4^, Marinus Vermeulen^4^, Liesbeth Siderius^5^

##### ^1^Oscar Nederland, Postbus 91, 4000 AB Tiel, The Netherlands; ^2^Paediatrician University of Kelaniya and Colombo North Teaching Hospital, Ragama, Sri Lanka; ^3^Consultant Paediatrician, Colombo, Sri Lanka; ^4^Rare Care World, Loosdrecht, the Netherlands; ^5^Paediatrician, Shwachman Support, Waddinxveen, The Netherlands

**Background**

Clinical prevention and treatment guidelines are essential to ensure the high standards of living for individuals with a rare and potential disabling condition. The need of a universal frame for a holistic care becomes more urgent. Countries differ in size, economy, population and health and social care systems. To support good data collection, interoperable information systems based on fixed standards need to be put in place [1].

Thalassemia, an inherited blood disorder due to a mutated haemoglobin gene, occurs across the globe [2]. Abnormal haemoglobin causes early destruction of red blood cells which manifests as anaemia, usually in childhood.

**Methods**

To study the applicability of universal standards, we compared thalassaemia care in two countries, one in Asia and one in Europe. Sri Lanka and the Netherlands are similar in population size but have different economy, size, and health system. Both countries offer a population screening program and recognize a medical care guideline. We aligned both the screening practice and guidelines with universal standards: Standard for identifying health measurements, observations, and documents (LOINC), International Classification of Diseases (ICD), International Classification of Functioning, Disability and Health (ICF), Anatomical Therapeutic Chemical Classification System (ATC), Clinical health terminology (SNOMED), Classification of rare diseases (ORPHA) and Catalogue of Human Genes and Genetic Disorders (OMIM).

**Results**

We identified a set of international standard terminologies for early diagnostic features of thalassaemia with diagnostics (LOINC, SNOMED) and therapies (ATC) for follow up. Disease terminologies can be defined by ORPHA, OMIM in combination with ICD. The ICD can be connected with the ICF, to document social impact of diseases. However, these terminologies are not used in either health system. Not in Sri Lanka, because healthcare data are still on paper, governed by the family. Nor in the Netherlands, where electronic records are governed by health care institutions and terminologies are implemented mostly for administrative reasons.

**Conclusions**

Standardization of collected data is needed to understand what measures can be taken to overcome health and social disparities. The use of international terminologies and standards in relation to guidelines are a tool for benchmarking quality of health care. At present there are insufficient or no incentives to stimulate the use of terminologies and interoperability standards for the improvement of care. Partnerships are needed between governments, healthcare providers, industry and patient organizations to develop a holistic framework of which all parties will benefit.

**Acknowledgement**

Soroya Beacher, patient expert, died on June 11th of the complications of Sickle Cell Disease.

**Reference**

1. Wyber R, Vaillancourt S, Perry W, Mannava P, Folaranmi T, Celi LA. Big data in global health: improving health in low- and middle-income countries. Bull World Health Organ. 2015 Mar 1; 93(3): 203–208.

2. Modell B, Darlison M. Global epidemiology of haemoglobin disorders and derived service indicators. Bull World Health Organ. 2008; 86 (6): 480–487.

### P4 MalatiRari Live! The innovative video conferencing and video consultation system for rare disease patients. Rare professionals for the rare community

#### Renza Barbon Galluppi^1^, Romano Astolfo^2^, Enrico Capiozzo^3^, Serena Bartezzati^1^, Tommasina Iorno^1^

##### ^1^Uniamo F.I.M.R. Onlus, Rome; ^2^Sinodè Srl, Padova; ^3^Veasyt-spinoff Venice University Ca’ Foscari, Venice

###### **Correspondence:** Renza Barbon Galluppi

Malati Rari Live! https://live.malatirari.it/it/ is an innovative web platform that allows those suffering from rare diseases, or with a suspected rare disease, to get in touch and talk with a network of professionals, right from the comfort of their own home. This is possible thanks to a video conferencing and video consultation system involving professionals from different areas: doctors and specialists, psychologists and consultants, admin staff, representatives of RD patient organizations, people affected by rare diseases and their relatives, people who have already gone through the experience of living with a rare disease thus able to help those who are at the start of the process. In particular, the service has three main focus: legal-administrative issues for rare patients and PO’s; psychological support; information on medical, social and health care.

MALATIRARI – VIDEO CONSULTATION is the result of a project submitted by UNIAMO FIMR Onlus (Uniamo Italian Non-profit Federation for Rare Diseases) to the “Digital for Social” call launched by the Vodafone foundation. UNIAMO's aim is to use this project to meet the need, emerged from various research projects, to receive correct and up-to-date information, together with the possibility to talk to people who have experienced the same situation and with qualified professionals. Available in 5 languages and other communication systems, the platform is available for international users, thanks to an innovative on-line video-interpreting service that can be also used by deaf people who use Italian sign language (LIS) thanks to a sign language interpreter. A large component of content is made with an alternative augmentative communication (CAA) to be as inclusive as possible. Psychological support is another important service offered by MalatiRari Live! in collaboration with SAIO, the Information and Orientation Service of UNIAMO FIMR Onlus which already responds to the toll-free number 800662541. The service will be available in form of teleconsultation on the platform to give psychological support to RD patients and caregivers that have to cope with the burden of worries, anxiety, anger and discouragement that they often experience. To access the video consultation service users must simply click on the REGISTER button in the top right-hand corner and select: Professional, Association, User, ePAG. Once the registration is completed the service is available. Patient representatives from European Reference Networks (ERNs) are also for part of the community. The interpreting service provided also opens the network up to international experts, respecting European guidelines on rare and complex diseases.

### P5 Development of an information pack to support families with children with Infantile Onset Pompe Disease to go to school

#### Vivienne Beckett^1^, Jane Lewthwaite^2^

##### ^1^Sanofi Genzyme, Oxford, Oxfordshire, OX4 2SU, UK; ^2^ssociation for Glycogen Storage Disease (UK) (AGSD-UK), Droxford, Southampton, SO32 3QY, UK

###### **Correspondence:** Vivienne Beckett (Vivienne.beckett@sanofi.com)

**Background**

Pompe disease, an ultra-rare genetic disorder, affects infants, children and adults. It impacts muscle with potential consequences for breathing, eating, sleeping, and mobility [1]. The classic infantile-onset form (IOPD) affecting children diagnosed under one year is severe and extremely rare [1]. Historically, most babies with IOPD died before their first birthday [1], however now children are reaching the age where they can go to school. The benefit of school extends beyond learning the curriculum - children can build relationships, make friends and gain confidence.

**Methods**

A Clinical Nurse Specialist at Great Ormond Street Hospital approached Sanofi Genzyme to look for ways to help more children with IOPD to experience and engage in a full primary educational life, whilst acknowledging parental concerns about how their child could safely thrive in this new and challenging environment.

Sanofi Genzyme coordinated a core steering group comprising a broad group of clinicians, parents whose children had made the transition to school, and the AGSD-UK to develop content to meet their needs. A comprehensive information pack was developed with sections for both parents and schools. The parent sections help families understand their rights and discuss with their chosen school the changes/adaptations that are needed to fully support their child’s development. The school sections of the pack were developed to help schools understand the impact IOPD has on a child and their family and how to support them. The pack was reviewed by both other UK specialist treatment centres that treat IOPD and was disseminated to parents by metabolic clinical nurse specialists, and via AGSD-UK.

**Results**

All families in the UK of school-age children with IOPD received the pack. Qualitative feedback on the resource pack highlights the value of the information to parents when engaging with schools about the needs of their children. Significant improvements in health-outcomes and the experiences of children have been documented through the use of these resources [2]. Unexpected positive outcomes include the education of other care professionals like physiotherapists and dentists about IOPD and that the pack supports application process for disability benefits. Metabolic nurses also report that the pack provides such a comprehensive resource that they can communicate more efficiently and effectively with education providers.

**Conclusion**

The pack has enabled families to obtain Education and Health Care Plans which provide a formal basis for extra care and support through school and results in parents feeling more confident about their children joining the education setting.

**Acknowledgements**

I would like to thanks Sindikwe Mnkandla, the clinical team at GOSH, the Pompe patient community, nurses at Manchester Children’s Hospital and Birmingham Children’s Hospital, and Aurora Healthcare communications.

**References**

1. Kishnani,P; Steiner, R; Bali, D; *et al.* Pompe Disease Diagnosis and Management Guideline. Genetics in Medicine. 2006; 8:267-288

2. Data on file, Sanofi Genzyme UK, 2018

### P6 Establishing the Norwegian Registry on Rare Disorders

#### Linn Grimsdatter Bjørnstad, Stein Are Aksnes

##### Norwegian National Advisory Unit on Rare Disorders, Oslo, Norway

###### **Correspondence:** Linn Grimsdatter Bjørnstad (linbj3@ous-hf.no)

Patient registries are considered as important tools for rare disorder surveillance. In Norway, a disorder is considered rare when there are less than 100 known cases per million inhabitants [1]. Currently, there is no nationwide record of rare disorder cases in Norway, and such knowledge is highly requested. The Norwegian National Advisory Unit on Rare Disorders consists of nine centres of expertise, each responsible for specific disorders, which provide services to ensure that people with rare disorders receive holistic and individually based care. In order for a service to be established for a rare disorder, the condition must meet the criteria of being congenital/of genetic origin, as well as being complex and compound. Furthermore, there must be a need for multidisciplinary and cross-institutional services throughout the course of life. In order to meet the needs of increased visibility and survey of rare disorders, the Norwegian National Advisory Unit on Rare Disorders has initiated the work of creating a national population-based patient registry, the Norwegian Registry on Rare Disorders [https://sjeldenregisteret.no]. Establishment of the registry is part of our 2017-2021 strategy and development of the registry is supported by the Norwegian Directorate of Health. An advisory board has been closely involved in the establishment process.

The primary objective of the registry is epidemiologic surveillance of rare disorders in Norway. This, in turn, will contribute to ensuring equity in services to patients with rare disorders. Furthermore, the registry will facilitate recruitment of patients to clinical trials and create a basis for quality assessment and research purposes. The registry will also serve as a basis for policy making and planning of health and social services for people with rare disorders.

The registry will be based on informed consent from the participants and, according to Norwegian legislation; a license from the Norwegian Data Protection Authority is required. The license was obtained in April 2018 and we propose to initiate data collection by the end of 2018. The registry will consist of core data, mainly comprising patient ID (national identification number) and information on demographics and diagnosis. We aim to create a registry that harmonizes with international standards. Data will be collected by health care professionals. Close collaboration with national health services and patient organisations will be of a crucial importance for a successful implementation of the Norwegian Registry on Rare Disorders.

**References**

1. Loeb, M, Grut L. [Rare disabilities in Norway. Need for knowledge about incidence and prevalence]. Report A9263. SINTEF rapport; 2008.

### P7 Autism spectrum disorder in patients with rare diseases

#### Adriana Bobinec, Ana-Maria Ivankov, Mijana Kero, Ivona Sansović, Ingeborg Barišić

##### Children's Hospital Zagreb, Medical School University of Zagreb, Zagreb, Croatia

**Background**

Autism Spectrum Disorder (ASD) is a heterogeneous group of neurodevelopmental disorders defined by impaired social-communication and presence of restricted, repetitive patterns of behaviour or interests. According to European Commission (2005), estimated prevalence of ASD in UK was between 10-30 per 10 000. The ASD has a strong genetic basis indicated by higher recurrence risk within affected families and in monozygotic vs. dizygotic twins, as well as the co-occurrence with chromosomal disorders and rare genetic syndromes.

**Objective**

Clinical and molecular analysis of ASD patients in order to identify patients with rare disorders associated with genomic copy number variants (CNVs).

**Methods**

Our study included 110 children referred to us with psychiatric diagnosis of ASD. Patient’s samples were screened for clinically relevant CNVs using chromosomal microarray in period from Jan 2016 until Jan 2018. We also analysed available parental samples.

**Results**

CMA analysis detected CNVs in 21 of total 110 patients studied. Pathogenic CNVs were found in 13 patients (Group A) while in additional 8 patients we detected variants of unknown significance (VUS) (Group B). Genetic changes found among patients in Group A included: 1p36 deletion (1), 2q33.1 deletion (1), 8p23.1 microdeletion (1), 15q11.2 microdeletion (2), 15q11-q13 duplication (1), 15q13.3 microdeletion (1), 17p11.2 deletion (1), 22q11.21 micro duplication (1) and 22q13.3 deletion (1). We also found small deletions associated with autism that affected region 2q21.1 (1) and genes *CNTNAP2* (1) and *NRXN1* (1). All patients in Group B had duplications that ranged from 131Kb to 1.6Mb. In one patient we found 16p13.11 micro duplication (1.6Mb) known to be associated with ASD and other variable phenotypic features. Pathogenicity of this CNV is unclear since it has also been found in healthy individuals. In 5 patients CNVs included genes involved in: neuronal development *FGF12* (2), *CNTNAP2* (1); axon guidance *PLXNA4* (1) and gene associated with X-linked mental retardation *BRWD3* (1) but since duplications of these genes have no evidence of pathogenicity, they have been categorized as VUS. In additional two patients we found CNVs in 1p13.3 and Xq26.2 regions that have been previously detected in patients with intellectual disability but their clinical significance is still uncertain.

**Conclusion**

Detailed clinical and diagnostic assessment should be performed in all patients presenting with autistic features. CMA analysis proved to be useful diagnostic tool in diagnosing rare genetic conditions in individuals with autistic spectrum disorder.

### P8 Qol-gNMD and patient reported functioning in genetic neuromuscular diseases: a new rasch tool

#### François Constant Boyer^1,2,3^, Amandine Rapin^1,2,3^, Antoine Dany^4^, Jean Benoit Hardouin^5^

##### ^1^Reims Champagne Ardenne NMD expert Center, Sebastopol hospital, 51092, Reims, France; ^2^Physical and Rehabilitation Medicine Department, Sebastopol hospital, 51092, Reims, France; ^3^EA 3797, Research associated team, Reims Champagne Ardenne University, 51095, Reims, France; ^4^Western Brittany University, Medicine and Health Sciences University, 29238, Brest, France; ^5^UMR 1246 INSERM – SPHERE “methodS in Patientcentered outcomes and HEalth ResEarch”, 44200, Nantes, France

**Background**

The “Quality of Life in genetic Neuromuscular Disease” questionnaire (QoL-gNMD) is a new patient reported outcome (PRO) measure tool specifically designed for patients with a slowly progressive neuromuscular disease with genetic predominant muscular damage. The QoL-gNMD has been developed in 3 domains: “Impact of Physical Symptoms”, “Self-perception” and “Activities and Social Participation”. The objective was to construct and develop a PRO tool, easy to use in clinical settings, and validated by rasch model theories.

**Methods**

Qol-gNMD was developed using focus groups consisting of expert patients meeting (5 focus group, n = 41, item bank) [1] and then a quantitative construction phase (n = 150, principal component and RMT analysis) [2]. The French version of the QOL-gNMD (26 items) was administered to patients recruited in 9 tertiary hospitals dedicated to genetic neuromuscular diseases (Fig. 1). Each QoL-gNMD domain is measured on a T score metric i.e. a normal distribution with a mean of 50 and a standard deviation of 10. High values represent good quality of life. Standard errors of measurement were estimated and adjusted using rasch model theories (RMT). For each QoL-gNMD domain we estimated, face validity, test retest reproducibility, concordant validity and the conditional minimum detectable changes [3].

**Results**

315 patients were recruited for psychometric assessment. Each domain showed good psychometric properties (person separation index > 0.7, test-retest ICC>0.7) and fitted the partial credit model (RMT) (Table 1). Concurrent validity was assessed using the WHOQOL-BREF. Estimated conditional MDC were calculated for each possible measure change.

**Conclusion**

QoL-gNND is an operational rasch validated measure. QoL-gNMD English version is available. We need psychometric validations of the English version and studies of psychometric properties for each pathology.

**Acknowledgements**

This multicentre project has been funded by the AFM Telethon (Association Française contre les myopathies) and the Champagne Ardenne area and Great East area of France. Thanks to Créteil, Angers, Nantes, Lille, Nice, Nancy expert centres to have participated at this project.

**References**

1. Dany A, Barbe C, Rapin A, Réveillère C, Hardouin JB, Morrone I, Wolak-Thierry A, Dramé M, Calmus A, Sacconi S, Bassez G, Tiffreau V, Richard I, Gallais B, Prigent H, Taiar R, Jolly D, Novella JL, Boyer FC. Construction of a Quality of Life Questionnaire for slowly progressive neuromuscular disease. Qual Life Res. 2015;24(11):2615-23.

2. Dany A, Rapin A, Réveillère C, Calmus A, Tiffreau V, Morrone I, Novella JL, Jolly D, Boyer FC. Exploring quality of life in people with slowly-progressive neuromuscular disease. Disabil Rehabil. 2017;39(13):1262-1270.

3. Dany A, Rapin A, Lavrard B, Saoût V,Réveillère C, Bassez G, Tiffreau V, Péréon Y, Sacconi S, Eymard B, Dramé M, Jolly D, Novella JL, Hardouin JB, Boyer FC. The quality of life in genetic neuromuscular disease questionnaire: Rasch validation of the French version. Muscle Nerve. 2017;56(6):1085-1091.

**Fig. 1 (abstract P8).**
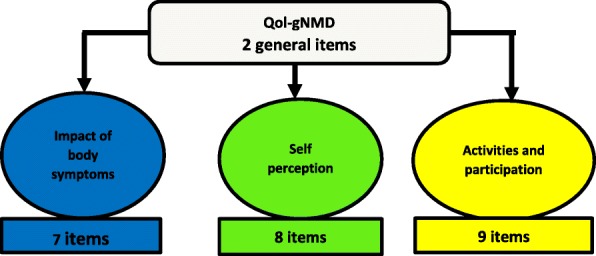
Qol-gNMD questionnaire

**Table 1 (abstract P8).** Psychometrics properties and Qol-gNMD questionnaire (n=315 patients)

	Impact of body symptoms	Self perception	Activities and social participation
Adequation test (Hi)	0.38	0.40	0.41
Person separation index (PSI)	0.77	0.81	0.85
Intraclass correlation (ICC)	0.75	0.79	0.80

### P9 Retina International

#### Avril Daly^1^, Roderigo Bueno^2^, Leighton Boyd^3^, Rosemary Boyd^3^, Laura Brady^4^, Markus George^5^, Tina Houlihan^6^, Brian Mansfield^7^, Kristin Smedley^8^, Christina Fasser^9^

##### ^1^Retina International, Dublin, Ireland; ^2^Retina, São Paulo, Brazil; ^3^Retina, Brisbane, Australia; ^4^Fighting Blindness, Dublin, Ireland; ^5^Pro Retina, Aachen, Germany; ^6^RP Fighting Blindness, Buckingham, UK; ^7^Foundation Fighting Blindness, Columbia, MD, USA; ^8^Curing Retinal Blindness Foundation, Ivyland, PA, USA; ^9^Retina International, Zurich, Switzerland

Retina International is a global umbrella group of 43 patient led member organisations who collaborated with a Scientific and Medical Advisory Board, Geneticists, young retinal researchers and media specialists to develop a fully accessible On-line Patient Toolkit on Genetic Testing. The Toolkit was rolled out in 2017 and was supported by a dedicated communications campaign. The objective of this unique multi-stakeholder project is to provide educational tools to patients and medical professionals that will enable coordinated global advocacy for equitable access to and reimbursement of Genetic Testing Services.

Inherited Retinal Diseases (IRDs) cause severe vision loss and the impact on quality of life is immense. Following decades of research potential treatments are now emerging. The genetic characteristics of IRDs mean a reliable genetic diagnosis is a prerequisite for inclusion in clinical trials. However, low levels of awareness among patients and medical professionals and issues of cost have resulted in a barrier to access. Retina International (RI) developed an on-line Genetic Testing Toolkit to educate stakeholders about the new possibilities of genetic diagnosis, providing tools to advocate for accessibility to and re-imbursement of genetic testing services nationally and internationally.

RI worked with its patient members, clinicians and researchers using focus groups, webinars and surveys to establish the level of knowledge in the first instances in each country and then among individual patients and their representatives. The surveys allowed for establishing the level of knowledge of the medical professionals with which the patients interact. Through a multi-stakeholder approach developed a consensus approach to inform a suite of accessible web based communications materials targeted at patient organisations and professionals in ophthalmology. RIs high level Scientific and Medical Advisory Board (SMAB) worked with a team of patient leaders drawn from seven countries and students in retinal research to collate the information generated and write content. A design team with expertise in accessible media are facilitating the production of simple user friendly downloadable materials including text, audio, and visual content for global application.

Working with these stakeholders to provide advocacy tools to ensure fair and equitable access to testing will assist in the populating patient registers and better facilitating the clinical trial process and will ultimately expedite innovation in the area of ophthalmology and empower patients through education and knowledge dissemination.

### P10 Case management at NoRo Centre in Romania (INNOVCare project)

#### Dorica Dan

##### RPWA (Romanian Prader Willi Association), Zalau, Romania

NoRo Center is a resource center and provides ”one stop shop services” for people with rare diseases from Romania.

It has been opened in 2011 through a Norwegian grant and a mentoring program with Frambu Resource Center for Rare Diseases in Norway.

NoRo has been accredited as a center of Expertise and together with 4 genetic centers from Timisoara, Iasi, Oradea and Craiova established the Ro-NMCA- ID (Romanian Network for Congenital Malformations and Intelectual Disabilities) and became member of ITHACA ERN.

NoRo continued to improve its services through case management under INNOVCare project (*4 case managers ½ FTE for 120 patients*) - I*nnovative Patient-Centered Approach for Social Care Provision to Complex Conditions:* https://www.innovcare.eu, funded by the EaSI programme of the European Union.

Objectives:

1. Developing holistic care for patients living with rare diseases in Romania;
2. Establishment of inter-sectorial and public-private partnerships for monitoring and assessment of the rare diseases patients needs and create networks in community to address their needs;

Eurordis has facilitated and supported the creation and governance of a European network of resource centres for rare diseases RareResourceNet, the European Network of Resource Centres for Rare Diseases.

Our case managers’ experience in this project revealed that in order to support even the most isolated patients we need a supportive network in the community, so that we started to train community nurses, social workers from the local authorities, teachers, nurses and other stakeholders on case management.

The case managers of NoRo Center concluded that the main support for our patients is needed in the areas of: Information about disease, information about their rights as a patient, self-management, communication skills, knowledge of available services, management of the disease, acceptance in community and coordination of care among stakeholders;

We have promoted the case management as the work method of for community nurses in the National Strategy for Community Nursing and since the end of 2017, people with disabilities in our country has the right to have case managers according to the Social Assistance Law no. 292/2011 (1 case manager at 50 persons with disabilities).

**Conclusion**

Organizing supportive networks in the community, developing our resource center and exchanging best practices at national and international level create better access to care for patients.

### P11 The Cystic Fibrosis Community Advisory Board (CF CAB) – a transparent collaboration that puts the patient perspective into the heart of research, development and access to CF treatments

#### Hilde De Keyser (hilde.dekeyser@cf-europe.eu)

##### Cystic Fibrosis Europe (CFE), the European Federation of Patient organisations of people with Cystic Fibrosis, Brussels, 1160, Belgium

Over the last decades the importance of stakeholder collaboration in healthcare has become good practice all over Europe. CF Europe (CFE) has worked over the last 15 years to put the patient at the centre of CF research, drug development and discussions on access.

In 2016 CFE launched a new and innovative project, the Cystic Fibrosis Community Advisory Board (CF CAB), to do this on a European level in a structured, transparent and meaningful way. A Community Advisory Board (CAB) is a platform consisting of representatives of the patient community who meet with representatives of organisations such as companies and other research bodies to promote dialogue between patient communities and other healthcare stakeholders involved in research, development and treatment access discussions, to improve patients’ understanding of the science behind the disease and how treatments are brought to patients (e.g., the regulatory processes, pricing mechanisms, etc.) and to provide patient expertise on clinical trials and treatment design so patients’ needs are better met. The CF CAB is a way for patients to have an impact on all stages of therapeutic development, a means to ensure that the patient community’s voice is heard on topics of key importance for CF patients such as access to treatments, clinical trials and communications around them, as well as the future of CF research.

The CF CAB is a patient-led and patient-driven working group of CFE totally independent from any company or other organisation. The members of the CF CAB are nominated by the national CF patient associations, members of CF Europe, and approved by the CFE board. The CF CAB reports back to the CFE board. CFE guarantees good governance of the CF CAB through organizational support, training of the CF CAB members on key issues, managing the projects and networking. Much organisational support comes from the EURORDIS EUROCAB program. The CF CAB is a group of patients and patient representatives from 11 European countries and we will grow this group in the coming years. The CF CAB is in the process of looking to motivate other stakeholders to engage via the CF CAB, to perhaps include clinical trials networks and other associations.

### P12 Good off-label use practice (GOLUP) for patients with rare diseases

#### Marc Dooms^1^, Guy Goodwin^2^, TM van der Zanden^3,4^, SN. de Wildt^3,4^

##### ^1^University Hospitals Leuven, Leuven, Belgium; ^2^University Department Warneford Hospital, Oxford, United Kingdom; ^3^Erasmus MC Sophia Children’s Hospital, Rotterdam, The Netherlands; ^4^Department of Pharmacology and Toxicology, Radboud University, Nijmegen, The Netherlands

Off-label use of medicines is the practice of using a medicine outside of its authorized indications. In rare diseases, the use of medicinal products off-label is widespread and primarily driven by the lack of authorized medicines for specific indications. This was confirmed by a recent Commission’s study in this area, which identifies the large amount of rare diseases and the difficulty and high cost of carrying out clinical trials as the main reasons for the lack of authorized drugs.

While not optimal, off-label prescribing may remain essential to address unmet medical needs of patients with rare diseases. However, off-label practice, by definition, entails an increased risk for patients compared to medications that have gone through the authorization process. This is especially concerning because the manner in which countries deal with the off-label use of medicines is not harmonized across the European Union.

We believe that the below criteria, drawn together by independent experts, and stemming from decades of research and clinical practice, serve to provide a clear framework on when and how the off-label use of medicinal products can safely take place. They are applicable to the rare diseases field as well as to other areas where off-label use frequently occurs, such as paediatric care.

In light of the above, off-label use of medicinal products should only occur if all of the following criteria are met:

1. Presence of a medical therapeutic need based on a current examination of the patient by a suitably qualified health care professional;
2. Absence of authorized treatment and licensed alternatives tolerated by the patient or repeated treatment failure;
3. A documented review and critical appraisal of available scientific evidence favours off-label use to respond to the unmet medical need of the individual patient;
4. Patients (or their legal representative) must be given sufficient information about the medicines that are prescribed to allow them to make an informed decision;
5. Presence of established reporting routes for outcomes and adverse events linked to off-label use.

The identified criteria do not aim to limit off-label use, but seek to promote a harmonized approach for its occurrence in order to maintain the highest levels of patient safety and minimize adverse events.

**References**

1. Dooms M. The Challenges for the Prescriber and the Pharmacist: Off-label and Unlicensed Use. SIOPE’s Community Newsletter. 2011 Feb (6): 8-9.

2. Dooms M. Understanding off-label use and the new challenges. Orphanet Journal of Rare Diseases 2014, 9,1.

3. Dooms M, Cassiman D and Simoens S. Off-label Use of Orphan Medicinal Products: a Belgian Qualitative Study. Orphanet Journal of Rare Diseases 2016: 11 (1) 144.

4. Dooms M & Killick J. Off-label Use of Medicines: the need for good practice guidelines. Int J Risk Safety in Med 2017; 29 (1-2): 17-23.

5. https://ec.europa.eu/health/sites/health/files/files/documents/2017_02_28_final_study_report_on_off-label_use_.pdf

### P13 Why Respiratory Health in Duchenne Muscular Dystrophy (DMD) matters?

#### Vanessa dos Reis Ferreira (Vanessa.dosReisFerreira@santhera.com )

##### Patient Advocacy Europe, Santhera Pharmaceuticals, Pratteln, Switzerland, 4133

**Background**

Duchenne Muscular Dystrophy (DMD) is a rare and fatal muscle disease that causes muscle degeneration. Patients and family members with DMD have a challenging journey. Typically, patients living with DMD receive care from several providers and move regularly within health care settings, so high-quality transitional care is especially important for them, as well as for their family caregivers [1]. DMD patients and carers become the experts on their unique experiences living with the DMD condition. Patient engagement is an expanding area, elevating patients from solely research subjects to active partners along the development and lifecycle of medicines. A variety of methods that are used to engage patients have been described [2]. Accordingly, Santhera Pharmaceuticals has developed activities to engage with DMD patient groups.

**Methods**

An independent party built and conducted an online questionnaire to be carried out with DMD patients and family members or caregivers. The questionnaire was administered directly to 14 participants from 12 countries. Data were collected in October 2017. The questionnaire was administered through the software Survey Monkey and data were analysed using descriptive statistics.

**Results**

Respiratory problems are one of participants’ highest priorities for treatment. Problems associated with respiratory function decline have a high impact on patients with DMD as well as on their carers, which increases over time. Wide differences exist concerning the care available to people affected by DMD, and its quality, between and within European countries. While some countries have health care centres specialised in DMD, other countries have less than a hand full of specialists for DMD. Not all health care centres specialised in DMD have multi-disciplinary teams. In addition, transition from child to adult health services is not yet a standard procedure within certain countries. Considerable variability exists concerning the age at which children are first tested for respiratory problems, which ranges between 4 and 12 years. This has potential implications for interventions aimed at reducing respiratory function decline. Finally, people affected by DMD are not sufficiently informed about respiratory problems.

**Conclusions**

This study points out the need to improve the quality of respiratory care for people affected by DMD and to increase their access to information about respiratory problems.

**Acknowledgements**

We thank participants of the study, for completing the online questionnaire.

**References**

1. Ferrara L, Morando V, Tozzi V. The transition of patients with rare diseases between providers: the patient journey from the patient perspective. International Journal of Integrated Care. 2017;17(5):A157.

2. Domecq JP, Prutsky G, Elraiyah T, et al. Patient engagement in research: a systematic review. BMC Health Serv Res 2014;14:89.

### P14 Cooperation in the Field of Rare Diseases: A Social Science Perspective

#### Fanny Duysens (Fanny.Duysens@uliege.be)

##### Spiral Research Centre, University of Liège, Liège, Belgium

As unanimously claimed within the rare disease community, cooperation between all stakeholders is a necessity in order to tackle these diseases and improve the difficult situations faced by patients and families. Public authorities, health professionals, scientists, industrials, or obviously patients and relatives’ associations, have an active part to play in the collective projects that are currently being developed worldwide. However, putting cooperation into practice does not always go unhindered.

Drawing on a qualitative research in social sciences, this poster presented some drivers and effects of cooperation in the field of rare diseases in Belgium and, more generally, in Europe. It highlighted some meaningful points of agreement and tension such as the degree of adequacy between international guidelines and national constraints, the confrontation between professional and experiential points of view, or the multiple meanings and uses of the “rare diseases” category. On the one hand, at an individual level, the analysis of some Belgian actors’ motivations and strategies for advocacy since the early 2000s showed the problematic accordance of their visions and claims due to different statutes, roles, and activities. On the other hand, turning to the collective process of elaboration of an action program within the context of the European Project for Rare Diseases National Plans Development (2008-2015), the positive outcomes of the constitution of a “trading zone” were pointed out , that is, a multi-stakeholders site that allowed managing communication despite divergent points of view and defining an homogenous “rare diseases” category [1]. Yet, regarding the dynamics of implementation of the Belgian Plan for Rare Diseases nowadays, the discrepancies between the stakeholders’ statutes within the healthcare system, as well as the constant (re)emergence of diseases and patients as singularities that is inherent to the approach of the whole, weaken the representativeness of all of them and the making of social links aligning to rare diseases issues. So the question remains open of the evolution of such trading zones at national and European levels. Are they likely to consolidate, even to institutionalize, or to fade away?

To conclude, the poster stated the value of multidisciplinary perspectives and mutual exchanges between social scientists and stakeholders, for the joint understanding of, and cooperation in, the lively field of rare diseases.

**Reference**

1. Galison P. Trading with the Enemy. In: Gorman ME, editor. Trading Zones and Interactional Expertise. Creating New Kinds of Collaboration. The MIT Press. Cambridge & London; 2010. p. 25–52.

### P15 Catalonia's Care Model for Rare Diseases (Spain)

#### Roser Francisco^1^, Pilar Magrinyà^1^, Josep Torrent^1^, Josep Jiménez^1^, Lluís Franch^1^, Àlex Guarga^1^, Diana Altabella^1^, Xavier Pérez^1^, Cristina Mallol^1^, Josefa Rivera^2^, Cristina Nadal^1^

##### ^1^Servei Català de la Salut (CatSalut), Ministry of health of Catalonia, Barcelona, Spain; ^2^Hospital Universitari Parc Taulí, Sabadell, Spain

###### **Correspondence:** Roser Francisco (rfrancisco@catsalut.cat)

People living with rare diseases (RD) are vulnerable to healthcare systems because standard procedures are not adequate to these patients. Catalonia has defined a care model by considering the viewpoint of RD’s patients [1], clinicians, professionals of administration bodies and the recommendations published by EUCERD [2] and the MSSSI [3]. It aims to optimize the available resources and improve care quality, integrating patient care levels and circuits that guarantee access to clinical expertise unit networks (XUEC).

**Key concepts of the Model** (Fig. 1)

- **Person-centred care.** Care is organized around the affected person and its family.
- **Equity of access**. Guaranties that every person affected by a RD has fast access to specialized care.
- **Expertise identification.** A XUEC per group of RD. Essential criteria: expertise, comprehensive care plan (diagnosis, treatment, monitoring), multidisciplinary team, case management, transition from paediatric to adult care and commitment to share knowledge.
- **Integrated care network.** It has two levels and implies a collaborative attitude and eHealth tools usage:
  - **expert care level (XUEC)** based on clinical expertise units (a network itself). It leads knowledge sharing, promotes teamwork and shares patient’s therapeutic and monitoring process with the territorial level.
  - **territorial/community level**, close to patient’s residence. Includes: primary healthcare, territorial hospitals, other health services, as well as social, educational and labour resources.
- **Catalan population registry**. Generation of RD high quality data from XUECs.

**Process of Implementation** (Fig. 2)

- **Thematic groups of RD**. RD (~7.000) are grouped in thematic groups (similar to ERN^3^ grouping).
- **By phases**. A few RD thematic groups will be prioritized and worked annually. In 2017, the first XUEC was designated; it addresses ***Genetic cognitive behavioural diseases***. Next XUEC call calendar: ***Hereditary metabolic disorders*** and ***Kidney diseases*** in 2018, ***Neuromuscular diseases*** and ***Immunodeficiency, auto inflammatory and autoimmune diseases*** during 2019.
- **Collaborative approach**. RD groups are prioritized in collaboration with the Catalan RD Expert Committee (integrated by patients, professional experts, and three Catalan Ministries -health, welfare and education-). A second committee constituted by representatives from 11 hospitals gives support.
- **Professional’s participation.** Call’s specific criteria are defined by experts in the field of the RD group. During 2017-2018 around 100 professionals have participated in the process.
- **Quality assessment.** A quality framework and periodic follow-ups assure expertise by establishing a dynamic model of evaluation (includes patients’ participation).

The model described hopes to resolve the main problems identified in the attention of RD by changing the traditional model towards a more **person-centred care**. The success of this model depends mainly in maintaining **dialog** and **collaboration** with patients and professionals, and the **engagement** of health care providers and CatSalut itself.

**Acknowledgements**

The Catalan RD Expert Committee, an advisory committee of the Ministry of Health of Catalonia (*Comissió Assessora de Malalties Minoritàries, CAMM*).

**References**

1. Kole A, Faurisson F, Mavris M. The voice of 12,000 patients: experiences and expectations of rare disease patients on diagnosis and care in Europe. Paris: Eurordis; 2009.

2. FEDER. Por un modelo sanitario para la atención a las personas con Enfermedades Raras en las Comunidades Autónomas. Estudio ENSERio. Madrid; 2013.

3. EUCERD. Workshop report: Centres of Expertise & European Reference Networks for Rare Diseases. Luxembourg: European Union; 2010.

4. Ministerio de Sanidad, Servicios Sociales e Igualdad (MSSSI). Estrategia en Enfermedades Raras del Sistema Nacional de Salud. Madrid: MSSSI; 2009.

**Fig. 1 (abstract P15).**
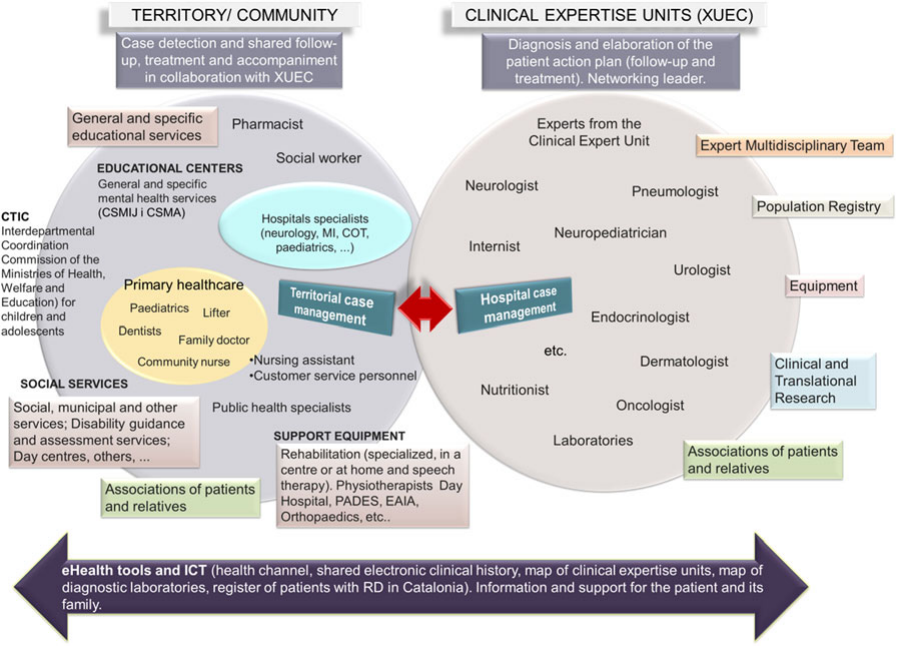
Catalonia's Care Model for RD. An integrated care network with two levels, an expert care level (XUEC) and a territorial/community level

**Fig. 2 (abstract P15).**
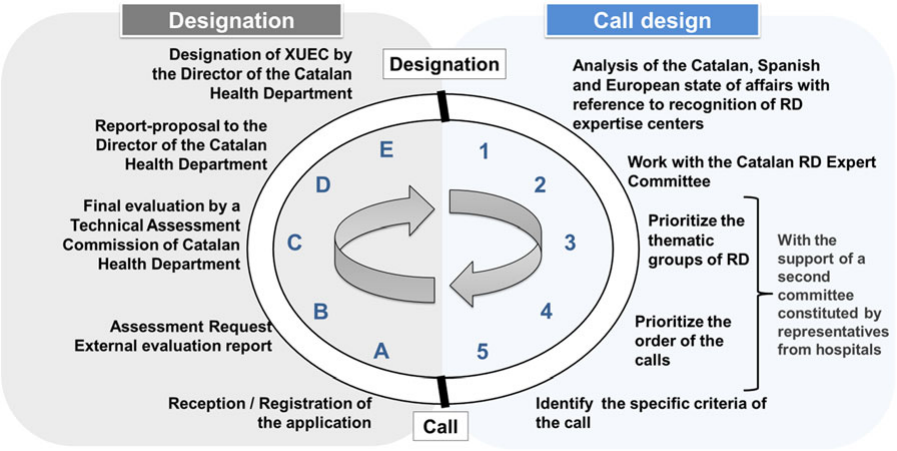
Implementation process. The project management is a cyclic and dynamic process

### P16 Ophthalmology outcomes in the treatment of von Hippel-Lindau syndrome with propranolol

#### Beatriz González-Rodríguez^1^, Karina Villar Gómez de las Heras^2^, Luis Rodríguez Padial^3^, Virginia Albiñana^4,5^, Lucia Recio-Poveda^4^, Ángel Cuesta^4,5^, Luisa María Botella^4,5^, Rosa María Jiménez Escribano^1^

##### ^1^Ophthalmology Department, Retina Service, Complejo Hospitalario de Toledo (SESCAM), Toledo, 45005, Spain; ^2^Pharmacy Department, Servicio de Salud de Castilla-La Mancha (SESCAM), Central Services, Toledo, 45071, Spain; ^3^Cardiology Department, Complejo Hospitalario de Toledo (SESCAM), Toledo, 45005, Spain; ^4^Centro de Investigaciones Biológicas CSIC, Madrid, 28040, Spain; ^5^Centro de Investigación Biomédica en Red de Enfermedades Raras (CIBERER), Madrid, 28029, Spain

###### **Correspondence:** Beatriz González-Rodríguez (glezrbeatriz@gmail.com)

Von Hippel-Lindau is a rare multisystem cancer syndrome caused by mutations of the VHL gene. Mutation carriers have a predisposition to develop benign and malignant tumours in different organs throughout their life, including retinal hemangioblastomas [1-9]. So far, there is no pharmacological treatment.

Retinal hemangioblastomas are the most common tumour and the earliest to give symptoms, causing retinal exudates, among others. Juxtapapillary and optic nerve hemangioblastomas are difficult and risky to be treated, and in most cases these patients lose their vision without any alternative [1-9].

Propranolol is a-non selective β-blocker whose safety has been demonstrated [11]. It’s the first-line treatment for infantile hemangioma[13, 14]; recent studies proved its use in breast cancer, melanoma, cavernous hemangiomas, and it may increase the efficacy of chemotherapy [15-21]. Albiñana *et al. in vitro* assays shown that *propranolol decreases HIF expression levels* and suggesting a *potential role of propranolol as vasoconstrictor, antiangiogenic agent (inhibiting VEGF), and proapoptotic drug (leading to cell apoptosis)* [10, 12].

To evaluate therapeutic effect of propranolol in VHL disease, a clinical trial including 7 VHL patients with juxtapapillary or peripheral hemangioblastomas was developed. Patients took 120 mg/day propranolol and were monitored at baseline and at 1, 3, 6, 9 and 12 months after. On every visit alongside the treatment, funduscopy as well as different biomarkers from blood samples were analysed. As the main clinical outcomes, number and size of tumours present on the retina remained stable and no new tumours appeared. To highlight, the reabsorption of the exudation in the only two patients who had it initially, being progressive and clear (Figs. 1 and 2). These outcomes correlated with the decreasing of VEGF plasma levels from the first month of treatment in a significant manner (p<0.001), reaching normal levels (<50 pg/ml) in all cases after 3 months of treatment. Moreover, a decrease in the expression of tumour stemness genes (*Sox-2*, *Oct-4)*, and proangiogenic genes (Epo and VEGF), an increase in proapoptotic gene Bax was found. These are the first biomarkers proposed to monitor the VHL disease activity [10, 12].

The results of this trial led to the orphan drug designation of propranolol by the European Medicines Agency EMA, to treat the VHL disease (EU/3/17/1841). Propranolol may be used in the future for diseases where VEGF is involved, so new trials are needed. *As peripheral blood levels of VEGF and miR210 correlated with retinal disease, they may be good biomarkers of the disease in the future* [10, 12].

**Trial registration**

Identifier 2014-003671-30

Protocol code: VHL-HOPE-2014-1

**Consent to publish**

All patients included in this clinical trial gave their consent to participate and be published.

**References**

1. Wong WT, Agrón E, Coleman HR, et al. Clinical Characterization of Retinal Capillary Hemangioblastomas in a Large Population of Patients with von Hippel-Lindau Disease. *Ophthalmology*. 2008. doi:10.1016/j.ophtha.2007.03.009.

2. Knudson AG. Cancer genetics. *Am J Med Genet*. 2002;111(1):96-102. doi:10.1002/ajmg.10320.

3. Gläsker S, Bender BU, Apel TW, et al. Reconsideration of biallelic inactivation of the VHL tumour suppressor gene in hemangioblastomas of the central nervous system. *J Neurol Neurosurg Psychiatry*. 2001;70(5):644-648. doi:10.1136/jnnp.70.5.644.

4. Bader HL, Hsu T. Systemic VHL gene functions and the VHL disease. *FEBS Lett*. 2012;586(11):1562-1569. doi:10.1016/j.febslet.2012.04.032.

5. Findeis-Hosey J, McMahon K, Findeis S. Von Hippel–Lindau Disease. *J Pediatr Genet*. 2016. doi:10.1055/s-0036-1579757.

6. Singh AD, Shields CL, Shields JA. von Hippel–Lindau Disease. *MAJOR Rev Surv Ophthalmol Surv Ophthalmol*. 2001. doi:10.1016/S0039-6257(01)00245-4.

7. Ben-Skowronek I, Kozaczuk S. Von hippel-lindau syndrome. *Horm Res Paediatr*. 2015;84(3):145-152. doi:10.1159/000431323.

8. Kanski JJ, Bowling B. *Kanski’s Clinical Ophthalmology. A Systematic Approach*. (Elsevier Health Sciences, ed.).; 2011.

9. Wong WT, Liang KJ, Hammel K, Coleman HR, Chew EY. Intravitreal ranibizumab therapy for retinal capillary hemangioblastoma related to von Hippel-Lindau disease. *Ophthalmology*. 2008;115(11):1957-1964. doi:10.1016/j.ophtha.2008.04.033.

10. Albiñana V, Escribano RMJ, Soler I, et al. Repurposing propranolol as a drug for the treatment of retinal haemangioblastomas in von Hippel-Lindau disease. *Orphanet J Rare Dis*. 2017. doi:10.1186/s13023-017-0664-7.

11. Flórez J, Armijo JA, Mediavilla Á. *Farmacología Humana*. Masson; 2001.

12. Albiñana V, Villar Gómez de Las Heras K, Serrano-Heras G, et al. Propranolol reduces viability and induces apoptosis in hemangioblastoma cells from von Hippel-Lindau patients. *Orphanet J Rare Dis*. 2015;10(1):118. doi:10.1186/s13023-015-0343-5.

13. Léauté-Labrèze C, Hoeger P, Mazereeuw-Hautier J, et al. A randomized, controlled trial of oral propranolol in infantile hemangioma. *N Engl J Med*. 2015;372(8). doi:10.1056/NEJMoa1404710.

14. Greenberger S, Bischoff J. Infantile hemangioma-mechanism(s) of drug action on a vascular tumor. *Cold Spring Harb Perspect Med*. 2011;1(1). doi:10.1101/cshperspect.a006460.

15. Powe DG, Voss MJ, Zänker KS, et al. Beta-blocker drug therapy reduces secondary cancer formation in breast cancer and improves cancer specific survival. *Oncotarget*. 2010;1(7):628-638. doi:10.18632/oncotarget.101009.

16. Pasquier E, Ciccolini J, Carre M, et al. Propranolol potentiates the anti-angiogenic effects and anti-tumor efficacy of chemotherapy agents: implication in breast cancer treatment. *Oncotarget*. 2011;2(10):797-809. doi:343 [pii].

17. Barron TI, Connolly RM, Sharp L, Bennett K, Visvanathan K. Beta blockers and breast cancer mortality: A population-based study. *J Clin Oncol*. 2011. doi:10.1200/JCO.2010.33.5422.

18. Ganz PA, Habel LA, Weltzien EK, Caan BJ CS. Examining the influence of beta blockers and ACE inhibitors on the risk for breast cancer recurrence: Results from the LACE cohort. *Breast Cancer Res Treat*. 2012;129(2):549-556. doi:10.1007/s10549-011-1505-3.Examining.

19. Zabramski JM, Kalani MYS, Filippidis AS, Spetzler RF. Propranolol Treatment of Cavernous Malformations with Symptomatic Hemorrhage. *World Neurosurg*. 2016;88:631-639. doi:10.1016/j.wneu.2015.11.003.

20. Berti I, Marchetti F, Skabar A, Zennaro F, Zanon D VA. Propranolol for cerebral cavernous angiomatosis: A magic bullet. *Clin Pediatr (Phila)*. 2014;53(2):189-190. doi:10.1177/0009922813492885.

21. De Giorgi V, Grazzini M, Benemei S, et al. Propranolol for Off-label Treatment of Patients With Melanoma Results From a Cohort Study. *JAMA Oncol*. 2017. doi:10.1001/jamaoncol.2017.2908.

**Fig. 1 (abstract P16).**
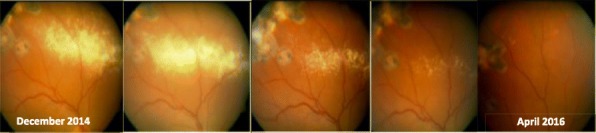
See text for description

**Fig. 2 (abstract P16).**
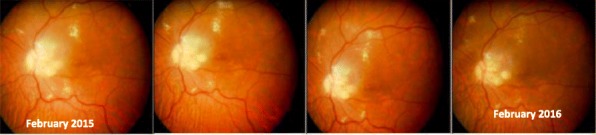
See text for description

### P17 healthbank - Your global people-owned health data transaction platform

#### Daniela Gunz, Rolf Eleveld, Karsten Stampa, Reto Schegg

##### Healthbank Cooperative, Blegistrasse 17a, 6340 Baar, Switzerland

###### **Correspondence:** Daniela Gunz

**Background**

healthbank is revolutionizing how personal healthcare data is exchanged, stored and monetized. Health data today is complex – it often is stored in closed ecosystems, sits dormant, is nearly never seen by the end user – the patient. Exchanging personal health data is difficult – patients and care providers rarely have a complete record of an individual. Health consumerism is driving how care is delivered and accessed. Patients increasingly have more data and choices at their fingertips and healthbank’s ecosystem enables users to securely store and exchange data with a range of healthcare, pharmaceutical and wellness and lifestyle service providers.

**Idea and Implementation**

healthbank was formed as a cooperative in 2013 in Switzerland. Our unique selling proposition (USP) is that the control and ownership of the data is solely with the user, which means that not only does healthbank come under the European Global Data Protection Regulation (GDPR) in terms of data protection, but it also represents a neutral and independent entity in the health data market.

Users, by giving consent, may share the collected data with other individuals or institutions (family members, physicians, caregivers, etc.) to optimize their own therapies and care. In addition, they can share their “real life data” for research purposes with research institutions and companies, anonymously and for an appropriate reward. The organizational model of a Swiss cooperative ensures that the data remain the property of and under the sole sovereignty of the user and only he or she may decide on the further use of the data.

**Call for Action**

Interested people can already create a free account on the healthbank platform [healthbank.me] and can support our journey by becoming a member of our healthbank cooperative [www.healthbank.coop]

**Fig. 1 (abstract P17).**
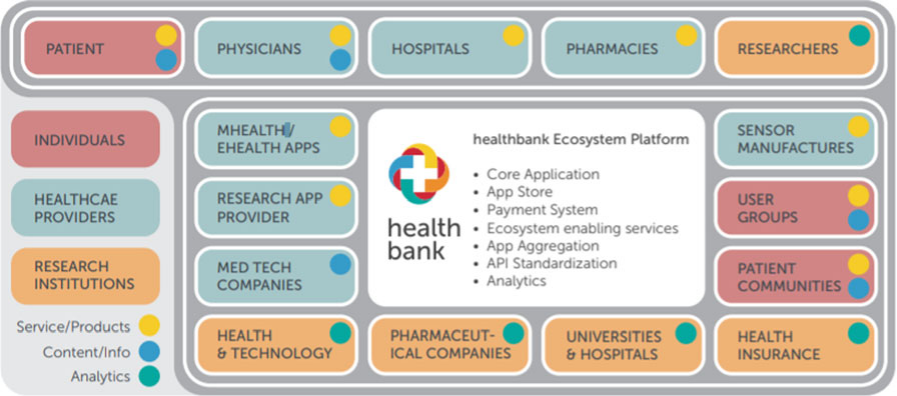
Healthbank ecosystem. Healthbank as a global platform is especially attractive for people with rare diseases. It provides a platform where people not only can host their medical and fitness data but also can search for and connect to people with the same symptoms; and in a second step, can contribute with their data to research and thus foster new therapies in fields with unmet medical needs

### P18 RD-Connect: an integrated infrastructure for data sharing and analysis in rare disease research

#### Dorota M Badowska^1^, Rachel Thompson^1^, Libby Wood^1^, Sergi Beltran^2,3^, Davide Piscia^2,3^, Steven Laurie^2,3^, Joan Protasio^2,3^, José María Fernández^4,5^, Rajaram Kaliyaperumal^6^, Séverine Lair^7^, Pedro Sernadela^8^, Marta Girdea^9^, , Volker Straub^1^, Marco Roos^6^, Peter A. C. 't Hoen^6^, Alfonso Valencia^4,5^, Lucia Monaco^10^, Chiuhui Mary P Wang^10^, Domenica Taruscio^11^, Sabina Gainotti^11^, Yllka Kodra^11^, Claudio Carta^11^, Paola Torreri^11^, David Salgado^12,13^, Christophe Béroud^12,13,14^, Hanns Lochmüller^1^, Ivo Gut^2,3^, the RD-Connect Consortium

##### ^1^John Walton Muscular Dystrophy Research Centre, Institute of Genetic Medicine, MRC Centre for Neuromuscular Diseases, Newcastle University, NE13BZ, UK; ^2^Centro Nacional de Análisis Genómico (CNAG-CRG), Center for Genomic Regulation, Barcelona Institute of Science and Technology (BIST), Barcelona, 08028, Spain; ^3^Universitat Pompeu Fabra (UPF), Barcelona, Spain; ^4^Centro Nacional de Investigaciones Oncológicas (CNIO), Madrid, Spain; ^5^Instituto Nacional de Bioinformática (INB), Madrid, Spain; ^6^Department of Human Genetics, Leiden University Medical Center, Leiden, The Netherlands; ^7^Interactive Biosoftware, Rouen, France; ^8^DETI/IEETA, University of Aveiro, Aveiro, Portugal; ^9^Centre for Computational Medicine, Hospital for Sick Children and University of Toronto, Toronto, ON, Canada, M5G0A4; ^10^Fondazione Telethon, Milan, 20121, Italy; ^11^National Rare Diseases Centre, Istituto Superiore di Sanità, Rome, 00161, Italy; ^12^Aix-Marseille Université, Marseille, 13391, France; ^13^Inserm, UMR_S 910, Marseille, France; ^14^APHM, Hôpital TIMONE Enfants, Laboratoire de Génétique Moléculaire, Marseille, 13005, France

###### **Correspondence:** Ivo Gut (ivo.gut@cnag.crg.eu)

RD-Connect [rd-connect.eu] is an infrastructure for rare disease research bringing together multiple data types in three systems [1, 2]: Genome-Phenome Analysis Platform, Registry & Biobank Finder and Sample Catalogue (Fig. 1). All of them are open to any rare disease and available free of charge.

The Genome-Phenome Analysis Platform [https://platform.rd-connect.eu] is a centralized data repository and a user-friendly online analysis system combining omics data (genomics, proteomics, transcriptomics) with clinical information at individual-patient, family or cohort level. Whole-genome, exome and gene panel datasets are submitted by the end-user and processed by a standardised analysis and annotation pipeline to make data from different sequencing providers comparable. Raw data is deposited at the European Genome-phenome Archive for long-term storage. Clinical information is recorded in the PhenoTips system, which simplifies entry of clinical data using the Human Phenotype Ontology. Results are available to the submitter and authorised users through the highly configurable platform, which enables advanced filtering and prioritization of variants. Users can analyse their own patients and compare results with other submitted cohorts, including queries such as: “Does this variant exist in this cohort?” and “Are there patients in other databases with matching phenotype and variants in the same gene?”. The Platform already includes thousands of datasets from partner projects such as NeurOmics [https://rd-neuromics.eu/] and BBMRI-LPC [www.bbmri-lpc.org]. In 2018, it became the primary data sharing and analysis platform for the Solve-RD project [http://solve-rd.eu/], which will bring in 19,000 unsolved cases from European Reference Networks over 5 years. RD-Connect is free and open for contributions from individual research groups and other projects.

The Registry & Biobank Finder [3] [http://catalogue.rd-connect.eu/] is a directory or rare disease patient registries and biobanks. For each resource, it provides contact data, numbers of patients/samples available for each disease and documentation.

The Sample Catalogue [https://samples.rd-connect.eu] allows browsing biosample collections stored in rare disease biobanks using powerful filtering functions. It provides detailed information about individual biosamples, including disease, diagnosis type, sample type, sex, availability of genetic and registry data and of samples from the patient’s relatives. Currently, the Sample Catalogue includes over 25,000 samples stored by rare disease biobanks in the EuroBioBank Network [4].

The work is ongoing to interconnect the three systems, to allow queries such as finding biosamples from patients with a specific genetic variant and identifying registries that hold their data.

**Acknowledgements**

RD-Connect Consortium has received funding from the European Union Seventh Framework Programme (FP7/2007-2013) under grant agreement No. 305444.

**References**

1. Lochmüller H & Badowska D, Thompson R, Knoers N, Aartsma-Rus A, Gut I, Wood L, Harmuth T, Durudas A, Graessner H, Schaefer F & Rieß O. RD-Connect, NeurOmics and EURenOmics: Collaborative European Initiative for Rare Diseases. Eur J Hum Genet 2018; 26(6): 778–785.

2. Thompson R, Johnston L, Taruscio D, Monaco L, Béroud C, Gut IG et al. RD-Connect: an integrated platform connecting databases, registries, biobanks and clinical bioinformatics for rare disease research. J Gen Intern Med 2014; 29(Suppl 3): S780–87.

3. Gainotti S, et al. The RD-Connect Registry & Biobank Finder: a tool for sharing and integrating aggregated data and metadata among rare disease researchers’ data sharing and data integration in rare disease research. Eur J Hum Genet 2018; 26(5):631-643.

4. Mora M, Angelini C, Bignami F et al: The EuroBioBank Network: 10 years of hands-on experience of collaborative, transnational biobanking for rare diseases. Eur J Hum Genet 2015; 23: 1116-1123.

**Fig. 1 (abstract P18).**
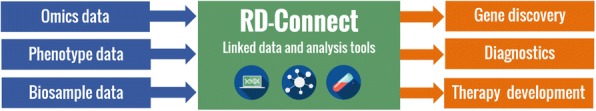
The RD-Connect infrastructure facilitates rare disease research by connecting different types of data into a common resource

### P19 Rare Academy – Sjelden.no

#### Kari Hagen^1^, Lene S Gloslie^2^, Elisabeth F. Bækken^2^, Rasmus S Dinessen^2^, Bjørn-Magne Stuestøl^2^

##### ^1^Frambu Resource Centre for Rare Disorders, Norway; ^2^The Norwegian Advisory Unit on Rare Disorders (NKSD), Norway

###### **Correspondence:** Kari Hagen

The Norwegian Advisory Unit on Rare Disorders (NKSD) established an open online academy in 2017. The target group is professionals working with people with rare disorders.

**Background**

Research and experience shows that when professionals lack knowledge, it makes life more challenging for people with a rare disorder.

**Goal**

Sjelden.no's goal is to effectively increase the knowledge about rare disorders amongst professionals by providing easy access, high-quality and relevant information.

**Target group**

Sjelden.no's target group is professionals working with people with rare disorders and their families. This includes professionals working in all sectors (government/private, health, school, kindergarten, special services etc.)

**Staff**

Sjelden.no consists of three fulltime staff members (editor, course developer and multimedia consultant), who collaborates closely with the nine centres for rare disorders in Norway. The staff is located at Frambu Resource Centre for Rare Disorders.

**Digital learning resources**

Sjelden.no offers a variety of different digital learning resources such as e-learning courses, videos, podcasts and lectures. In collaboration with the Inland Norway University of Applied Sciences, Sjelden.no is also developing a course on rare diseases giving 10 ECTS. The first students will enrol in the autumn of 2019.

**High quality content**

The resources are developed, and quality assured by the leading experts on rare disorders in Norway.

**Learning outcome**

Due to most professionals’ busy schedules, Sjelden.no is striving to maximize the learning outcome in a time efficient manner. Sjelden.no does this by incorporating learning methods that have been documented to give the best learning outcome.

**Feedback**

“I now have a much deeper understanding and feel more prepared for difficult situations that can arise” - Health professional

“As parents to a child with Neurofibromatosis, we are grateful that you have made an e-learning course about the diagnose targeting school professionals. Our dialogue with the school are now much better and productive. The school professionals have an increased and up-to-date knowledge about the diagnose and various challenges our child faces” - Father

### P20 Patients experiences are essential for improving the quality of health care

#### Marinda J. A. Hammann, Marije C. Effing-Boele, Hanka K. Dekker

##### Patient Organisation for Metabolic Diseases (VKS), Zwolle, The Netherlands, 8031 GJ

###### **Correspondence:** Marinda J. A. Hammann (info@expertiseinkaart.nl)

**Background**

Most patients with rare diseases (RD) feel they do not receive the attention they deserve. ‘Expertise Mapped’ was therefore set up to visualise the organisation of care for RD from the patients’ perspective (Fig. 1). Part of the developed method (based on EUCERD Quality Criteria) consists of an online survey to gather the patients’ perspective on health care. The whole method is explained on the website Expertise Mapped [https://www.expertisemapped.org]. From the collected data we have distilled common interests that can be used to advocate the needs of patients with RD.

**Method**

Between 2013 and 2017 1802 Dutch patients with 191 different RD filled in the online survey. Distinctive characteristics and similarities were identified and analysed with the one-way ANOVA and Paired Samples T-Test.

**Results**

28.7% of patients indicates to experience problems regarding their care. The greatest issue is the lack of knowledge among healthcare professionals regarding their disease (57.5%, Fig. 2). Overall, patients experience most difficulties with healthcare professionals other than their general practitioner or medical specialist (p<0.05).

Problems identified by the entire group are: insufficient support regarding the organisation/coordination of care (29.5%) and insufficient psychological support (36.1%). Of this latter group, 50.0% indicates a need for psychological support, 46.8% of whom would like this to be offered by the specialist at the university medical centre.

Of all respondents, 44.0% is aware of the existence of a centre of expertise (CE). 53.7% is willing to travel to always see an expert.

**Conclusion**

Despite the introduction of government-designated CEs in The Netherlands in 2014, less than half of the patients is aware of their existence. CEs should thus increase their visibility for patients, emphasize their added value and involve patients in the organisation of care, possibly with the method of ‘Expertise Mapped’.

Our data show that most patients are willing to travel to an expert. When a larger proportion of patients is seen in a CE, the knowledge of RD will increase and the quality of care will improve. Simultaneously, experts must pass along their expertise to other healthcare professionals to decrease the knowledge gap. Support for patients in the organisation/coordination of care is also important and should be provided by someone from the CE. This person should also stimulate the collaboration between different healthcare professionals and have attention for psychological support. All these data suggest there is a larger role for the CEs to fill the gaps that patients experience.

**Acknowledgements**

The project ‘Expertise Mapped’ is a collaboration between different patient organisations and together we published knowledge maps for 26 different RD. It is funded by the Dutch Government (FondsPGO). The Patient Organisation for Metabolic Diseases (VKS) is project leader. The project partners for 2018 are the Dutch patient organisations for Pulmonary Fibrosis, Vasculitis, Pulmonary Hypertension, Aplastic Anaemia & Paroxysmal Nocturnal Hemoglobinuria, Adrenal Gland Diseases (NVACP/BijnierNET), Anorectal Malformation, Fabry Support & Information Group and Tall People. Other patient organisations we collaborated with are the Dutch patient organisations for Amyloidosis, Bladder Extrophy, Congenital Melanocytic Nevus, Kidney Diseases, Ichthyosis, Osteogenesis Imperfecta, Phospholabam Gene Mutation (Harteraad), Short Bowel Syndrome.

**Fig. 1 (abstract P20).**
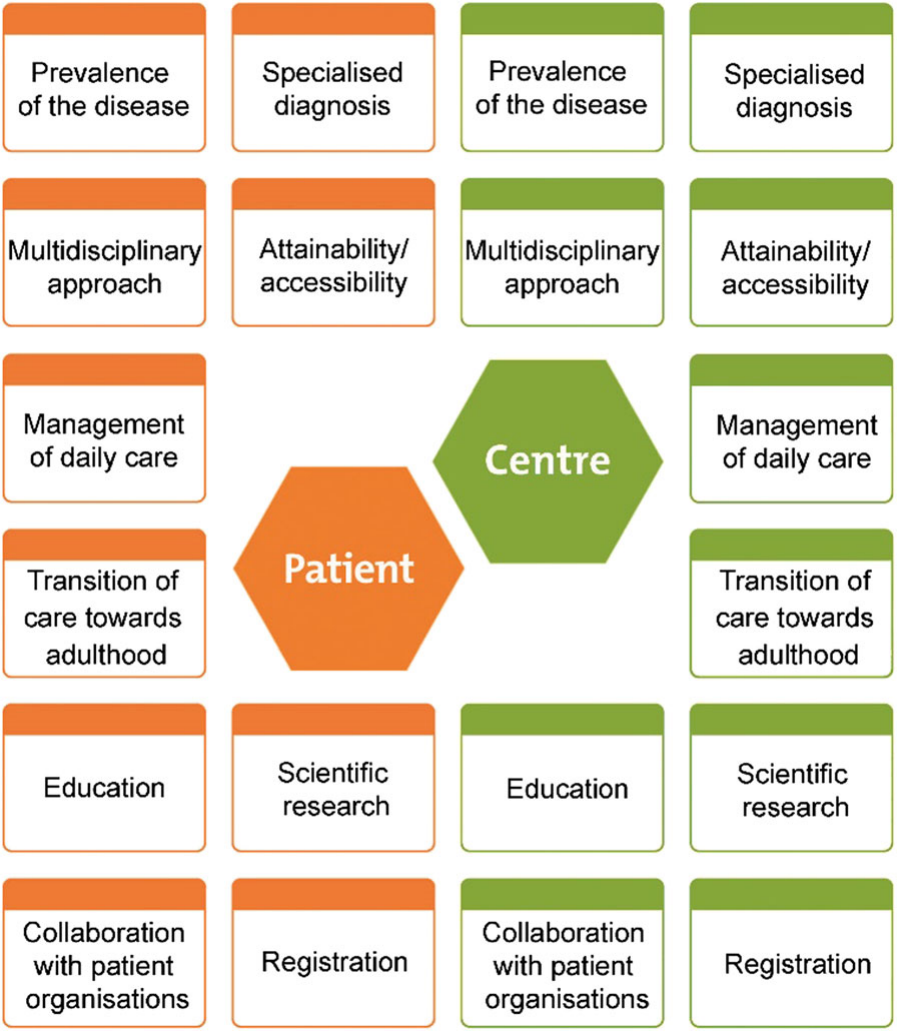
Example of a knowledge map developed by ‘Expertise Mapped’ to visualise the organisation of care for RD. The knowledge map shows the patients’ perspective on health care (gathered from an online survey, orange part) and the organisation of care in the CE (gathered from interviews with the experts by a panel of patients, green part)

**Fig. 2 (abstract P20).**
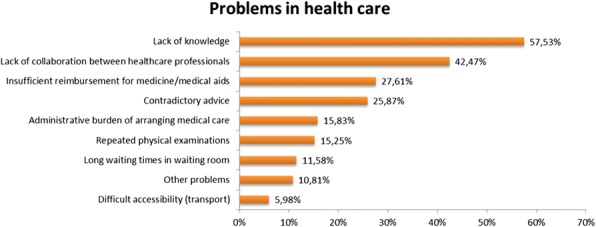
Patients are asked to identify the problems in health care they experience (n=518)

### P21 Finnish HAE patients’ experiences of quality of life – a survey

#### Risto Heikkinen (risto.heikkinen@allergia.fi)

##### Finnish Allergy, Skin and Asthma Federation, Helsinki, Finland

**Background**

The most characteristic signs of HAE are recurrent attacks of severe swelling. At worst the attacks can be lethal in case no proper medicine is available. The aim of the survey was to acquire more knowledge of the impact of Finnish HAE on patients’ quality of life and to develop the services and support provided for the HAE patients. Two surveys were conducted in 2017 - 2018.

**Results**

Almost half of the respondents had waited for the diagnosis over eight years or more after first symptoms and doubts. During the process, many patients experienced negative attitudes towards themselves and lack of support.

The impact of HAE on patients’ life varies a lot. Half of the respondents evaluated their quality of life poor. On the other hand, almost half of them found the impact of HAE very low. The role of the relatives was important for over half of the respondents. The non-predictable and possibly lethal nature of attacks caused stress to both patients and relatives.

During the last five years over half of the respondents have experienced a lot of individual variation in functionality. Over 40 % of the respondents estimate the negative impact of HAE being high. The respondents described how the non-predictable nature of attacks made their life difficult. Over half of the respondents claimed that HAE had affected their working career a lot. Also prejudices from the part of co-workers and employers are commonly reported in the open-ended questions. 40 % of the respondents evaluated that HAE had reduced and restricted their social life a lot. Most of the patients comment that with the help of medicine the quality of social life has improved.

Most patients have their follow-up organized in specialized health care units. Still, many have never met hospital’s social worker. Everyone was familiar with medical expenses reimbursement, but other social benefits and services are known only to some of them.

**Conclusions**

Health care professionals should pay more attention to the impacts of HAE on everyday life. Social workers should be always involved when evaluating patients’ needs for support. Further the role of relatives should better be taken into consideration in order to ensure the needed support.

Health care professionals should be better informed about the impact of HAE on patients. Further the co-operation with health and social care professionals should be enforced, especially within the Finnish ERN Skin Centre in Helsinki University Hospital.

### P22 Lessons learned from IDeAl — 33 recommendations from the IDeAl-Net about design and analysis of small population clinical trials

#### Ralf-Dieter Hilgers^1^, Malgorzata Bogdan^2^, Carl-Fredrik Burman^3^, Holger Dette^4^, Nicole Heussen^1^, Mats Karlsson^5^, Franz König^6^, Christoph Male^7^, France Mentré^8^, Geert Molenberghs^9^, Stephen Senn^10^

##### ^1^Department of Medical Statistics, RWTH Aachen University, Aachen, 52074, Germany; ^2^Institute of Mathematics and Computer Science, WROCLAW UNIVERSITY OF TECHNOLOGY, Wroclaw, 50-372, Poland; ^3^Department of Mathematical Sciences, Chalmers University of Technology, Göteborg, 41296, Sweden; ^4^Mathematical Statistics, Ruhr-University Bochum, Bochum, 44870, Germany; ^5^Department of Pharmaceutical Biosciences, Uppsala Universiteit, Uppsala, 75124, Sweden; ^6^Center for Medical Statistics, Informatics, and Intelligent Systems, Medical University Vienna, Vienna, 1090, Austria; ^7^Department of Paediatrics and Adolescents, Medical University Vienna, Vienna, 1090, Austria; ^8^Biostatistical Modelling and Pharmacometrics, French National Institute of Health and Medical Research, Paris, 75018 France; ^9^Interuniversity Institute for Biostatistics and statistical Bioinformatics, Universiteit Hasselt & KU Leuven, Hasselt, 3500, Belgium; ^10^Luxembourg Institute of Health, Luxembourg, 1445, Luxembourg

###### **Correspondence:** Ralf-Dieter Hilgers (rhilgers@ukaachen.de)

**Background**

IDeAl (Integrated designs and analysis of small population clinical trials) is a European Union funded project within the 7th Framework Programme [FP7 2007-2013, Ga. No 602552] developing new statistical design and analysis methodologies for clinical trials in small population groups. IDeAl follows strictly the concept of an improved integration of design, conduct and analysis of clinical trials from various perspectives. With the presented poster we provide a synergistic view of IDeAl findings over the past four years.

**Method**

The challenges to design and analyse small population clinical trials are described in [1]. The project’s currently 80 publications in peer-reviewed journals to date are used to derive recommendations linking the nine scientific IDeAl work packages adaptive design, biomarkers, decision theory, extrapolation, genetic factors, optimal design, pharmacogenetics, randomisation and simulation. The recommendations are related to various aspects of planning and analysis of a small population clinical trial.

**Results**

The developments of the IDeAl work packages are summarized in 33 recommendations from an applied viewpoint, giving researchers a comprehensive guidance to the improved methodology (see Table 1). In particular, the findings will help to design and analyse efficient clinical trials in rare diseases with limited number of patients available.

IDeAl established a new perspective on design and analysis of small-population clinical trials by a huge number of options to refine the statistical methodology for small-population clinical trials from various perspectives. Details can be found in [2].

**Conclusion**

Contrasting the 33 recommendations developed to the particular practical clinical situation the researcher is able to tailor the design and analysis of his small population clinical trial. The route to improvements is demonstrated by the IDeAl-network representing the unique new statistical methodological skills necessary to design and analyse small-population clinical trials. Training of biostatisticians and physician is necessary to apply the methods for improvement of clinical research in rare diseases.

**References**

1. Hilgers, RD., Roes, KC. and Stallard, N. Directions for new developments on statistical design and analysis of small population group trials. Orphanet Journal of Rare Diseases. (2016). 11 (1); 78

2. Hilgers, RD, Bogdan M., Burman CF., Dette H., Karlsson M., König F., Male C., Mentré F., Molenberghs G. and Senn S. Lessons learned from IDeAl — 33 recommendations from the IDeAl-Net about design and analysis of small population clinical trials. Orphanet Journal of Rare Diseases (2018) 13:77, 10.1186/s13023-018-0820-8

**Table 1 (abstract P22).** 33 recommendations for the design and analysis of innovative small population group clinical trials [2]

**Decision Theory – Carl-Fredrik Burman** R 1. Formulate decision rules in a formal Bayesian decision-theoretic framework. R 2. Societal decision rules (regulation, reimbursement) should be determined based on explicit modelling of how they will inter-depend with commercial drug developing decisions. R 3. Increase transparency in regulatory and payer decisions. R 4. The well-being of the individual trial patient must have priority. **Simulation of Clinical Trials – Mats Karlsson** R 5. If fast computations of power curves are needed from a non-linear mixed effects model, we recommend using the parametric power estimation algorithm as implemented in the stochastic simulation and estimation tool of PsN. R 6. The simulation methods described above can be utilized to investigate the effects of using different, smaller, more parsimonious models to evaluate data from complicated biological systems prior to running a clinical study. R 7. We recommend the use of Sampling Importance Resampling to characterize the uncertainty of non-linear mixed effects model parameter estimates in small sample size studies. Non-estimability of parameters may be assessed using preconditioning. The use of the bootstrap model averaging method is recommended when conducting model-based decision-making after a trial. Robust model-based adaptive optimal designs may be used to improve model certainty in clinical trials. **Optimal Design – France Mentré** R 8. For evaluation of designs of studies with longitudinal discrete or time-to-event data, evaluation of the Fisher Information matrix should be done without linearization. Using the new approach MC-HMC (in the R package MIXFIM) will provide adequate prediction of standard errors and allow to compare several designs. R 9. When there is little information on the value of the parameters at the design stage, adaptive designs can be used. Two-stage balanced designs are a good compromise. The new version of in the R functions PFIM can be used for adaptive design with continuous longitudinal data. R 10. When there is uncertainty in the model regarding the parameters, a robust approach across candidate models should be used to design studies with longitudinal data. **Genetic Factor Identification – Malgorzata Bogdan** R 11. It is recommended to use “varclust” for clustering of gene expression or metabolomics data and extraction of a small number of potential predictors of patients’ response to the treatment based on highly dimensional “omics”. Also, it is recommended to use PESEL for estimation of the number of important principal components. R 12. It is recommended to use both regular and group SLOPE for identification of biomarkers based on the genotype data, since regular SLOPE has a higher power of detection of additive gene effects, while group SLOPE allows for identification of rare recessive variants. R 13. It is recommended to use the modified Bayesian Information Criterion for efficient aggregation of genotype and ancestry of genetic markers and identifying biomarkers in admixed populations. **Surrogate Endpoints Evaluation – Geert Molenberghs** R 14. In case of small trials, which are in particular variable in size, we recommend the use of the causal inference framework, combined with efficient computational methods. R 15. In case of the evaluation of surrogate endpoints in small trials subject to missingness, we recommend the use of pseudo-likelihood estimation with proper inverse probability weighted and doubly robust corrections R 16. In case of hierarchical and otherwise complex designs, we recommend using principled, yet fast and stable, two-stage approaches. R 17. In case of genetic and otherwise high-dimensional markers, we recommend the use the methodology expressly developed for this context, in conjunction with the software tools made available (R package IntegratedJM). R 18. In case of a surrogate with dose-response or otherwise multivariate information present, we recommend to use the Quantitative Structure Transcription Assay Relationship framework results. R 19. In case of the evaluation of surrogate endpoints in small studies, we recommend using weighting-based methods, because the methodology has been shown to work well theoretically, it has been implemented in user-friendly software, and its practical performance is fast and stable. **Assessment of Randomization – Ralf-Dieter Hilgers** R 20. Do not select a randomisation procedure by arbitrary arguments, use scientific arguments based on the impact of randomisation on the study endpoint taking into account the expected magnitude of bias. R 21. Tailor the randomisation procedure used in small-population randomized clinical trial by following ERDO using randomizeR. R 22. In case of a randomized clinical trial, we recommend to conduct a sensitivity analysis to examine the impact of bias on the type-I-error probability. **Adaptive Design – Franz König** R 23. In the case of confirmatory testing, we recommend adapting the significance level by incorporating other information, e.g. using information from drug development programs in adults for designing and analyzing pediatric trials. R 24. Where randomized control clinical trials are infeasible, we propose “threshold-crossing” designs within an adaptive development program as a way forward to enable comparison between different treatment options. R 25. In the case of design modification during the conduct of a confirmatory clinical trial, we recommend using adaptive methods to ensure that the type-I-error is sufficiently controlled not to endanger confirmatory conclusions. Especially in clinical trial with multiple objectives special care has to be taken to address several sources of multiplicity. **Pharmacogenetics – Stephen Senn** R 26. For the analysis of N-of-1 trials, we recommend using an approach that is a modified fixed-effects meta-analysis for the case where establishing that the treatment works is the objective, and an approach through mixed models if variation in response to treatment is to be studied. R 27. When conducting a series of N-of-1 trials we recommend paying close attention to the purpose of the study and calculating the sample size accordingly using the approach provided in detail in Senn. R 28. We recommend that response should not be defined using arbitrary and naïve dichotomies but that it should be analysed carefully paying due attention to components of variance and where possible using designs to identify them. R 29. When analyzing between-patient studies, we recommend avoiding information-destroying transformations (such as dichotomies) and exploiting the explanatory power of covariates, which may be identified from ancillary studies and patient databases. **Extrapolation – Holger Dette** R 30. The comparison of dose response curves should be done by the bootstrap approach. R 31. If the aim of the study is the extrapolation of efficacy and safety information, we recommend considering and comparing the MEDs of two given populations. R 32. The derived methodology shows a very robust performance and can be used also in cases where no precise information about the functional form of the regression curves is available. R 33. In case of planning a dose-finding study comparing two populations, we recommend to use optimal designs in order to achieve substantially more precise results.	

### P23 Mental health and rare diseases: mixed methods study and policy recommendations

#### Rosa Spencer-Tansley, Amy Hunter

##### Genetic Alliance UK, CAN Mezzanine, 49-51 East Road, London, N1 6AH, UK

###### **Correspondence:** Amy Hunter (amy.hunter@geneticalliance.org.uk)

**Objective**

Patients and carers living with rare conditions frequently report poor emotional wellbeing and mental health but this has not been studied before. This study was designed to: (1) document the impact of living with, or caring for someone with, a rare condition, and experiences of health and other support services in relation to mental health; and (2) develop recommendations for improvement in mental health support for patients and carers.

**Method**

This was a mixed methods study with UK participants recruited through Rare Disease UK, Genetic Alliance UK and SWAN UK (syndromes without a name). Semi-structured interviews with sixteen patients and carers were transcribed and analysed thematically. The identified themes, and findings from the literature, were used to develop an online survey. The survey was disseminated through the same networks. Statistical analysis of 1,921 responses was mainly descriptive. The interview and survey findings led to specific recommendations for improvement which were refined through consultation at a stakeholder workshop. Stakeholders included representatives of patient organisations, professional organisations and health services, and individual clinicians, patients and carers. The report was launched in May 2018 [1].

This work was supported by a multi-disciplinary advisory group. The study was approved by the Social Care Research Ethics Committee.

**Results**

1. Living with a rare condition can have a huge impact on emotional wellbeing and mental health, with respondents reporting anxiety, stress, emotional exhaustion and suicidal thoughts. Despite this, positive coping experiences are still possible for many.
2. Some of the drivers of poor mental health reflect issues that are especially significant for conditions that are rare. This includes low levels of awareness of rare conditions among healthcare professionals; poor coordination of care leaving the patient or carer as ‘project manager’; difficulties in trying to get a diagnosis for the rare condition; and patients or carers not always being believed when trying to access care for physical health issues.
3. Our data have led to recommendations for improvements in practice through empowering individual healthcare professionals, and for service-level coordination of care to include mental health support [1].

**Conclusions**

Emotional wellbeing and mental health are a serious issue for individuals living with a rare condition. The specific drivers of poor mental health, and knock-on effects on people’s lives, are many and varied, and often reflect challenges that are particular to rare conditions. We have proposed a series of specific evidence-based recommendations for improvement in support.

**Acknowledgements**

This study was funded by public donations to Rare Disease UK, a campaign run by Genetic Alliance UK. We thank all the patients and carers who took the time to share their experiences, the workshop attendees and advisory group members for their input and expertise, and Mind – the mental health charity – for their support throughout the project.

**References**

1. Rare Disease UK. Living with a rare condition: the effect on mental health. London, UK; Rare Disease UK; May 2018. 24 p. Available from: https://www.raredisease.org.uk/our-work/living-with-a-rare-condition-the-effect-on-mental-health-2018/

### P24 Moving towards new rare disease research goals: IRDiRC defines its roadmap for 2018

#### Anneliene H Jonker^1^, Christine M Cutillo^2^, Lilian PL Lau^2^, Marlene Jagut^1^, Ana Rath^1^, Hugh JS Dawkins^3^, Christopher P Austin^2^

##### ^1^IRDiRC Scientific Secretariat, INSERM-US14, Paris, France; ^2^National Center for Advancing Translational Sciences (NCATS), National Institutes of Health, Bethesda, MD, USA; ^3^Department of Health Western Australia, Perth, Australia

###### **Correspondence:** Anneliene H Jonker (anneliene.jonker@irdirc.org)

Capitalizing on the momentum of progress made in rare diseases research over the past six years, the International Rare Diseases Research Consortium (IRDiRC) devised a new set of global rare disease goals for the upcoming decade. Announced in August 2017 [1, 2], IRDiRC aims to push the limits of what is currently possible in the longer term with an ambitious vision for the field, all with patients’ lives in mind:

*Vision: Enable all people living with a rare disease to receive an accurate diagnosis, care, and available therapy within one year of coming to medical attention* **.**

In order to work towards this vision, IRDiRC has set three goals for the next decade:

1. *All patients coming to medical attention with a suspected rare disease will be diagnosed within one year if their disorder is known in the medical literature; all currently diagnosable individuals will enter a globally coordinated diagnostic and research pipeline*
2. *1000 new therapies for rare diseases will be approved, the majority of which will focus on diseases without approved options*
3. *Methodologies will be developed to assess the impact of diagnoses and therapies on rare disease patients*

To tackle these goals, following extensive and collective discussion and prioritization, the Consortium defined its new roadmap for 2018 which includes actions to accelerate research and development in rare diseases, and to remove numerous barriers and bottlenecks. These actions are aimed at improving research funding and coordination, identifying strategies to diagnose unsolved cases, facilitating data collection and sharing, advancing therapy development, boosting patient engagement in research, and enabling international collaborations. IRDiRC Committees and Task Forces will deliver a series of recommendations for policies and standards, tools and services, and adoption of best practices in response to the needs of the rare disease community.

**Acknowledgement**

The abstract is presented on behalf of the International Rare Diseases Research Consortium (IRDiRC). CPA contributed to this work in his capacity as Chair of IRDiRC, not as Director of NCATS.

**References**

1. Austin CP, Cutillo CM, Lau LPL, Jonker AH, Rath A, et al. Future of Rare Diseases Research 2017-2027: An IRDiRC Perspective. Clin Transl Sci. 2018 Jan;11(1):21-27.

2. Austin CP, Dawkins HJS. Medical research: Next decade’s goals for rare diseases. Nature. 2017 Aug 9;548(7666):158.

### P25 Helpline Seltene Krankheiten – improving patient care in rare diseases

#### Saskia R. Karg^1,3^, Sabrina Strebel^1^, Damaris Hubacher^1^, Giatgen Spinas^2,3^, Matthias R. Baumgartner^1,3^

##### ^1^Division of Metabolism, University Children’s Hospital Zurich, Zurich, CH-8032, Switzerland; ^2^Division of Endocrinology, Diabetes and Clinical Nutrition, University Hospital Zurich, Zurich, CH-8091, Switzerland; ^3^radiz – Rare Disease Initiative Zurich, CRPP, University of Zurich, Zurich, CH-8006, Switzerland

###### **Correspondence:** Saskia R. Karg (saskia.karg@kispi.uzh.ch)

**Objective**

To improve care for rare disease patients in the German-speaking part of Switzerland.**Method**

Existing expertise in rare diseases was harnessed by building an interdisciplinary network, coordinating existing resources, and making resources visible and hence accessible. A phone and email helpline was established in order to facilitate patient access to resources, experts and expertise.

**Results and Conclusion**

In rare diseases, patients are rare, experts are rare and knowledge is fragmented. The unmet need is high, as is the burden on patients. Switzerland is a small country with 8 million inhabitants in four language regions. The number of patients affected by each of the many rare diseases is small and the different languages precipitate the problem. The hospitals of the University Medicine Zurich provide care for many rare disease patients.

Rare disease helplines have been established in many countries to help address the challenges faced by patients [1]. In Switzerland, a helpline serving the French-speaking region was successfully set up in 2014. Since 2016 the “Helpline Seltene Krankheiten” is serving the largest language region of Switzerland, the German-speaking part.

The free phone and email helpline serves people affected by rare diseases, their loved ones, as well as professionals looking for information such as referrals to resources and experts. A special emphasis is being placed on social resources, patient organizations, and any resources that further patient empowerment.

Help is being requested for more adult patients than pediatric patients, which may point towards a higher need in adult care. Clinical experts were most often sought, which due to the small size of Switzerland may sometimes only be found abroad. Patients also look for contact with other patients and we have been able to connect patients within Switzerland and abroad. For rare diseases where there is an active network of experts and / or patient organizations, the helpline usually does not receive inquiries. Patients without a diagnosis were referred to the undiagnosed patient program at the University Hospital Zurich. An effort will be made to make the helpline better known, but it remains unclear how patients who are unaware of the “rare disease label” and patients with lower internet and health literacy can be reached.

**References**

1. Houyez F, Sanchez de Vega R, Brignol TN, Mazzucato M, Polizzi A. A European network of email and telephone help lines providing information and support on rare diseases: results from a 1-month activity survey. Interact J Med Res. 2014; 3(2):e9

### P26 100,000 Genomes Project at Birmingham Children’s Hospital a start of genomics in re-shaping the diagnosis landscape for rare diseases

#### Maria Kokocinska, Larissa Kerecuk, Sharon Parkes, Jessica Kainth, Emily Hunter

##### Birmingham Women’s and Children’s Foundation Trust, B4 6NH, Birmingham, UK

###### **Correspondence:** Maria Kokocinska (Maria.Kokocinska@nhs.net)

**Background**

Our understanding of human DNA is ever expanding with over 500,000 genomes sequenced already [1]. Genomic information obtained from next generation sequencing analysis is already entering clinical practice by providing diagnosis for rare disorders. Furthermore, our advancing understanding and knowledge of human DNA together with innovations in genomic technologies are empowering decision making in healthcare.

**Objective**

The 100,000 Genomes Project (100kGP) is a continuation of research efforts in the field of genomics with the ambitious objective of implementing genomics medicine into the mainstream NHS as part of patients’ routine care [2, 3] for the first time in history in the world delivering personalised medicine. It is a truly ground-breaking project of NHS transformation attempting to deliver a precision medicine giving the right treatment to the right patient at the right time and at the right dose.

**Local approach**

Birmingham Children’s Hospital (BCH) started recruiting rare disease patients to 100kGP in June 2015. As a leading specialist paediatric centre, it is offering an expert care to children and young people from across the country with 270,000 visits every year. The award-winning, world-renowned hospital has a strong team of 3,700-staff and great reputation for excellence in many life-changing specialist services. The genomics team at BCH consist of 3 Research Coordinators and a Genomics Assistant led by the Rare Disease Lead (Consultant Nephrologist). The team has developed a streamlined process which incorporates recruitment to Project at the same time as patient’s routine appointment. BCH is the highest single contributor of recruits in the UK outside London having consented well over 3000 participants. As a result, the team are able to offer all identified, eligible patients the chance to become part of the project without increasing the work burden for the consultant or the number of appointments for the family; with excellent feedback from both groups.

**Conclusion**

The approach used at BCH to recruit patients and families affected by rare and undiagnosed diseases to the 100kGP shows that implementing genomics medicine into the mainstream NHS as part of patients’ routine care is possible. Patients and their families are very keen to take part. Clinical and scientific expertise will be employed in data analysis and validation and it is hoped that the results from the project will lead to new diagnosis, personalised management and improved outcomes for the patient and the NHS.

**Acknowledgements**

Many thanks to all the participating families with rare disease without them it would not be possible.

**References**

1. Herper M. Illumina Promises To Sequence Human Genome For $100 -- But Not Quite Yet. Forbes-Pharma & Healthcare 2017 JAN 9, 2017.

2. Genomics England. Narrative- GENOMICS ENGLAND AND THE 100,000 GENOMES PROJECT. 2016; Available at: https://www.genomicsengland.co.uk/the-100000-genomes-project/. Accessed 04/24, 2017.

3. Peplow M. The 100 000 Genomes Project. BMJ 2016;353(i1757).

### P27 “Ask and you will receive (Matthew 7:7)”; The experience of A.B.C. (Associazione Bambini Cri du chat) Italy

#### Maria Elena Liverani^1^, Maura Masini^2^, Kodra Yllka^3^, Marta De Santis^3^, Marianna Spunton^4,^ Andrea Guala^4^

##### ^1^UOC Pediatria, Ospedale Sant’Andrea, Roma, Italy; ^2^Associazione Bambini Cri du Chat (A.B.C.), San Casciano in Val di Pesa, Firenze, Italy; ^3^Centro Nazionale Malattie Rare (CNMR) - Istituto Superiore di Sanità (ISS), Rome, Italy; ^4^SOC Pediatria, Ospedale Castelli, Verbania, Italy

It was April 2016 when some articles about costs and quality of life were published on Eur J Health Econ, a sort of “provisional report” of BURQUOL-RD. The main aim of BURQOL-RD was to develop a disease based model (instrument) to assess the impact of health policies, interventions and treatments in the field of rare diseases (RDs) quantifying the Economic Burden and Health-Related Quality of Life (HRQOL) for patients and their caregivers, from a macro societal perspective. Ten RDs were targeted in the pilot study: Cystic Fibrosis, Prader-Willi Syndrome, Haemophilia, Duchenne Muscular Dystrophy, Epidermolysis Bullosa, Fragile X Syndrome, Scleroderma, Mucopolysaccharidosis, Juvenile Idiopathic Arthritis and Histiocytosis. For the first-time scientists tried to face the problem of costs of a rare disease. Not only direct costs, nor only health related costs, but also social, indirect and intangible costs.

For the first-time patients and caregivers were asked to answer questions about their health problems and to weigh the impact of the disease on their quality of life.

The cost-of-illness (COI) approach was used. Information about the patients’ and caregivers’ HRQOL was estimated using the generic EQ-5D questionnaire; besides, the patients’ disability was assessed through the Barthel Index and Whodas 2.0.

The diseases studied were different one form the other, and their prevalence was quite high, but we thought to ask the ISS (Istituto Superiore di Sanità=National Centre for Rare Diseases, Istituto Superiore di Sanità (ISS) Rome Italy) if it could be possible to figure something similar tailored on our problem: The Cri du chat syndrome.

It took time to adapt questionnaires without altering them (so that the comparison with “the original” could be possible) and to convince families to fill them (even if boring and time wasting), but in the end we obtained a pretty high participation and a huge amount of data which are now being processed by economists.

Already in this ECRD we have the possibility to read preliminary results, which will be precious to allocate resources and help policy makers and other stakeholders to plan wisely.

Our Association had already published in 2012 an article on Minerva Pediatrica on “*how much having a child with CdC Syndrome had modified parent’s life*” (*), but this new approach takes into account all different aspects of Quality of Life, and the answer comes from caregivers (very seldom Cri du chat patients are able to fill questionnaires themselves), not from “professionals”. We want to point out that even “small” RD can obtain tailored investigations if the proper path is found.

RD require the combined efforts of health and social care professionals, politicians, managers and researchers to increase the availability of effective disease management tools to improve care and to extend both life expectancy and Health Related Quality of Life (HRQOL).

We thank ISS for their sensibility and hope to have opened a new form of collaboration between associations and institutions, patients and doctors and policy makers. Just asking….

### P28 Orphacodes’ use for the codification of rare diseases: results of the testing activity carried out within the RD-Action framework

#### Monica Mazzucato^1^, Paola Facchin^1^, Elisa Salamanca ^2^, Celine Angin^2^, Claude Messiaen^2^, Feriel Framdi^2^, Stefanie Weber^3^, Magdalena Marx^3^, Franzisca Dulas^3^, Annie Orly^4^, Ana Rath^4^

##### ^1^RD Coordinating Centre-Registry, Veneto region, Italy; ^2^APHP Banque Nationale de Données Maladies Rares, Paris, France; ^3^DIMDI German Institute of Medical Documentation und Information, Cologne, Germany; ^4^Orphanet-Inserm US14, Paris, France

###### **Correspondence:** Monica Mazzucato

**Background**

The EC issued a Recommendation in 2014 [1] on how to improve the identification of RD using Orphacodes (OC), developed from the Orphanet classification [2]. Specific activities within the Joint Action for RD (2015-2018) [3] aimed at providing the necessary tools and guidelines to promote the adoption of OC within healthcare information systems across Europe.

**Methods**

Developed guidelines and coding resources have been object of a testing activity, divided into two

phases. The first phase consisted in a coding exercise, performed using data coming from Veneto region (Italy) and from France, where information systems monitoring RD patients are ongoing since 2002 and 2007, respectively. The analysis performed refers to the period 2007-2016 and to patients with a confirmed diagnosis. Results refer to the first part of the testing activity. The aim is to compare the real world use of OC in these two different settings and to evaluate the impact of the use of OC in terms of comparability of the monitored RD entities and of the monitored RD patients.

**Results**

The retrospective analysis of OC used in the Veneto region RD registry (VRRDR) and in the National French RD registry (BNDMR) involved 5,349 OC and 98,112 RD patients. Most of the OC belong to the “rare genetic diseases” branch (27% in VRRDR and 26% in BNDMR), followed by the “rare developmental anomalies” branch (14% in VRRDR and 17% in BNDMR) and the “rare neurological diseases” branch (12% in VRRDR and 10% in BNDMR). When considering existing patients, in the BNDMR, they are mainly described by OC belonging to the following groups: genetic RD (20%), rare developmental anomalies, neurological RD (9%), eye RD (7%). In the VRRDR patients’ distribution per OC is the following: genetic RD (22%), neurological RD (10%), eye RD (9%), rare developmental anomalies (8%). The differences can be due to the primary sources of data considered: mainly genetic Centres in France and both genetic and clinical Centres in VR. The comparability of patients’ distribution increases when considering the hierarchical structure of the classification, especially at the medium levels.

**Conclusions**

OC are suitable for use in different settings, showing a high level of comparability, especially if the hierarchical organization and the multidimensionality aspect of the Orphanet classification are preserved. Further issues that should be addressed deal with the level of granularity of the Orphanet classification that has to be considered to achieve better data comparability across MS and how the hierarchical and multidimensional aspect of the Orphanet classification could be exploited to serve RD coding purposes.

**References**

1. CEGRD (2014) Recommendation on ways to Improve Codification for Rare Diseases in Health Information Systems. Adopted at the 3rd meeting of the Commission Expert Group on Rare Diseases (12-13 November 2014). http://ec.europa.eu/health/rare_diseases/docs/recommendation_coding_cegrd_en.pdf

2. Orphanet (2017) Orphadata. http://www.orphadata.org/cgi-bin/inc/product1.inc.php

3. RD-ACTION: http://www.rd-action.eu

### P29 Paediatric Challenges in Orphan Drug Development, a Review of Issues and Possibilities Following 10th Anniversary of the European Paediatric Regulation

#### Camille Métais^1^, Frédéric Honoré^2^

##### ^1^Regulatory Affairs, Alexion Pharma GmbH, Zurich, Switzerland; ^2^Regulatory Affairs, Alexion Pharmaceuticals Inc., Boston, MA, USA

###### **Correspondence:** Frédéric Honoré (Frederic.Honore@alexion.com)

**Background**

Ten years after the implementation of the European Paediatric Regulation, new medicinal products development in children continues to present some challenges in particular in orphan diseases. A review of available data (EMA publications) relative to Paediatric Investigation Plans (PIPs) for orphan products was performed to identify the possible hurdles and to suggest potential ways of improvement.

**Results**

Around 50% [1] of all PIPs approved (2010-2014) postponed their completion by a median of 2.2 years. To date ~13% [1] of PIPs managed to get to completion and 45% [2] of these completed PIPs failed to obtain a reward. A total of 150 PIPs [3] have been agreed for orphan medicinal products (OMPs), with around 5% [3] (8) completed so far. The main difficulty for conducting studies in children in accordance with PIP requirements comes from recruitment issues (39% [3]) and the low numbers of paediatric patients in orphan disease further increases the issue. A broader use and acceptance of innovative approaches such as adaptive designs and extrapolation strategies may help to achieve effective developments. Suggestions for Paediatric regulation evolutions, identified based on the analysis, are displayed on Fig. 1.

Finally, PDCO recommendations and feedback may sometimes be disconnected with the feedback from local Health Authorities (HA) and Ethics Committees (EC) when implementing the agreed paediatric studies. A change in local HA and EC mind-set from “protect children from research” to “protect children through research” is may assist in facilitating conduct of paediatric developments.

**Conclusion**

The Paediatric Regulation translated into a positive impact on paediatric development of new drugs^3^. However, only few PIPs agreed for OMPs have been completed, and only three OMPs have obtained an extension of the market exclusivity period thanks to successful completion of PIP requirements. Companies developing products in rare diseases are already committed to conducting trials in children, as a large proportion of rare diseases are childhood diseases, but recruitment can be a barrier to PIP completion especially with the additional challenges associated with rare diseases. Rewards linked to PIP completion for OMP may need to be re-evaluated to better fit the current landscape.

More flexibility in the PIP regulatory framework and procedural simplifications could help in making sure that children’s medical needs are at the forefront of new OMPs development.

**References**

1. Hwang TJ, Tomasi PA, Bourgeois FT (2018) Delays in completion and results reporting of clinical trials under the Paediatric Regulation in the European Union: A cohort study. PLoS Med 15 (3): e1002520. H

2. Questions and Answers on 10 years of the EU Paediatric Regulation, 26 Oct 2017

3. European Commission 10-year report on Paediatric Regulation.

**Fig. 1 (abstract P29).**
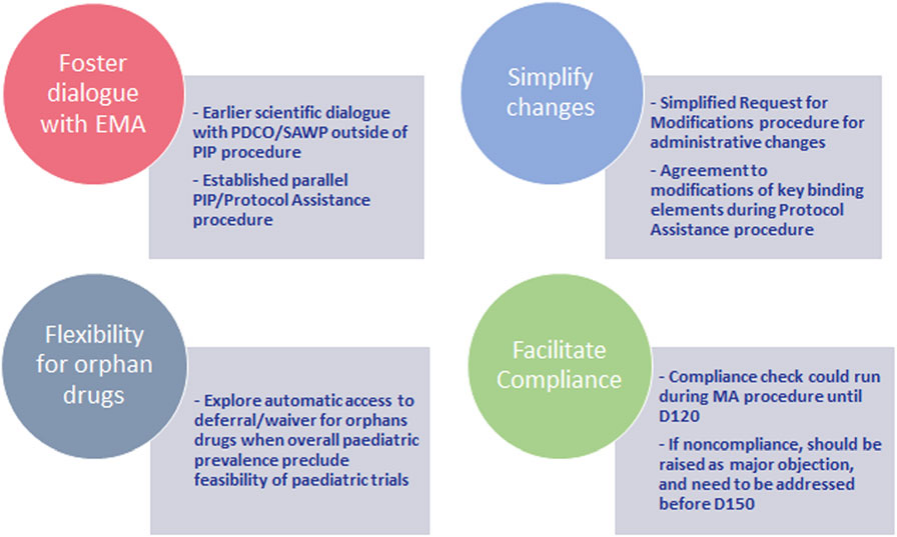
Potential for Improvements of European Paediatric Regulatory Framework

### P30 Gene therapy for ADA-SCID patients: Strimvelis as a successful model for the development of accessible advanced therapies for ultra-rare diseases

#### Lucia Monaco^1^, Michela Gabaldo^1,2^, Francesca Ferrua^2,3,4^, Maria Pia Cicalese^2,3^, Caterina Lucano^1^ and Alessandro Aiuti^2,3,4^

##### ^1^Fondazione Telethon, Milan, Italy; ^2^San Raffaele Telethon Institute for Gene Therapy, San Raffaele Scientific Institute, Milan, Italy; ^3^Pediatric Immunohematology and Bone Marrow Transplantation Unit, San Raffaele Scientific Institute, Milan, Italy; ^4^Vita-Salute San Raffaele University, Milan, Italy

###### **Correspondence:** Lucia Monaco (lmonaco@telethon.it)

Strimvelis, the first ex-vivo, autologous, stem cell gene therapy for adenosine deaminase severe combined immunodeficiency (ADA-SCID), is available today upon request to children lacking a suitable HLA-matched related donor.

This innovative medicine is the result of a 20 years’ research and development enterprise by the San Raffaele Telethon Institute for Gene Therapy in Milan, matured into the partnership with GlaxoSmithKline in 2010 that led to marketing authorization in Europe in 2016 (Fig. 1).

The eighteen children treated in the development phase were alive at three or more years’ post-treatment and did not undergo any serious adverse events; most had acquired immune competence [1, 2]. Four additional patients treated under expanded access and five children treated after the marketing authorization are showing outcomes in line with previous observations.

The therapy entails transduction of CD34+ cells from the patient’s bone marrow with a retroviral vector carrying the correct ADA gene. Cells are transduced in a clinical manufacturing facility nearby the hospital and reinfused fresh into the patient following low-dose chemotherapy. Overall, the treatment requires hospitalization for approximately six weeks and entails the presence of family members (parents and often siblings), given the patient’s very young age.

Today, Strimvelis is delivered exclusively at the San Raffaele Hospital in Milan, where all competences and structures required reside. All personnel have received specific training; a charitable program in support of the patients’ families during the treatment is in place. The Italian healthcare system set a price for the treatment that is the basis for agreements with national health systems and insurance bodies for cost coverage.

Overall, Strimvelis serves as an example of innovative therapy for an extremely rare disease made accessible to patients [3] that applies the best practices available at the time of its development. Therapy with Strimvelis is the starting model for the implementation of innovative gene therapy medicinal products and of new gene therapy production processes for other rare genetic diseases.

These could include, for example, the introduction of a freezing step in the preparation of the transduced cells. In all cases, only a few, specialised centres will be able to accrue the competences and facilities needed to offer advanced therapies such as Strimvelis to patients in an efficient and safe way.

While this and other solutions may contribute to enriching the portfolio of available gene therapies, Strimvelis will stay as an effective treatment benefitting children with ADA-SCID today and in the future.

### P31 “Mitochondrial DNA mutation *m.3243A>G*” – a phenotypic chameleon independent of age and gender

#### Katharina Niedermayr^1^, Gerhard Pölzl^2^, Sabine Scholl-Bürgi^1^, Christine Fauth^3^, Edda D Haberlandt^1^, Ursula Albrecht^1^, Wolfgang Sperl^4^, Johannes A Mayr^4^, Daniela Karall^1^

##### ^1^Department for child and adolescent health, Paediatrics I / III, Medical University of Innsbruck, Innsbruck, Austria; ^2^University Hospital for Internal Medicine III, Cardiology and Angiology, Medical University of Innsbruck, Innsbruck, Austria; ^3^Department of Medical Genetics, Molecular and Clinical Pharmacology, Human Genetics Division, Medical University of Innsbruck, Innsbruck, Austria; ^4^University Children's Hospital, Paracelsus Medical University Salzburg, Salzburg, Austria

###### **Correspondence:** Katharina Niedermayr (katharina.niedermayr@i-med.ac.at); Daniela Karall (daniela.karall@i-med.ac.at)

**Background**

In general, diagnosis of a mitochondrial disorder is based on symptom combinations and confirmed by genetic findings. However, patients carrying the *m.3243A>G* mutation in the mitochondrial tRNA-leucine 1 (MT-TL1) do not always meet all the proposed criteria for the most frequently encountered mitochondrial syndrome “MELAS”, which is an acronym for **m**itochondrial myopathy, **e**ncephalopathy, **l**actic **a**cidosis and at least one **s**troke-like episode. We here present various phenotypic characteristics of the mitochondrial mutation *m.3243A>G* with particular focus on the cardiac manifestations.

**Methods and results**

We followed nine patients (1 month to 68 years old; median 42 years; four female and five male) from nine different families with this *m.3243A>G* mutation in the mitochondrial tRNA-leucine 1 (MT-TL1). The classical “MELAS”-criteria are only met by three of these patients. Elektrocardiography shows preexcitation pattern with short PQ or rather PR intervals and delta waves (Wolff-Parkinson-White - WPW) in three patients and sick-sinus-syndrome plus atrioventricular block I° in one patient. Hypertrophic cardiomyopathy was found in eight patients with moderate to severe regurgitation of various valves.

**Conclusion**

Cardiac manifestation can encompass hypertrophic or dilated cardiomyopathy, as well as preexcitation syndromes or conduction delay. In general, the clinical presentation to fulfill the” MELAS”-criteria varies due to heteroplasmy. Thus, cardiologists should screen patients with unexplained cardiac features in the context of deafness, short stature and learning disabilities for mtDNA mutations, especially *m.3243A>G* mutation. A clear diagnosis is essential as a basis for prognostic advice concerning the disease course and clinical impact on family testing.

**Acknowledgement**

Original article is ahead of print in Congenital Heart Disease.

### P32 EUPID - General Data Protection Regulation (GDPR) compliant patient registration and pseudonymisation for Rare Disease Research

#### Michael Nitzlnader, Günter Schreier

##### Digital Health Information Systems, AIT Austrian Institute of Technology GmbH, Austria

###### **Correspondence:** Günter Schreier (guenter.schreier@ait.ac.at)

**Background**

REGULATION (EU) 2016/679 (General Data Protection Regulation - GDPR) came into effect as of May 25th, 2018 and brings new challenges in many areas where personal data are processed, including healthcare and research. The European Patient Identity (EUPID) Services are a set of web services, supporting independent, third party-based registration and pseudonymisation of patients. To prepare the roll-out of EUPID in the Rare Disease community, EUPID’s compliance with the GDPR requirements had to be assessed.

**Methods**

EUPID pseudonymisation results in a context-specific patient registration, i.e. one and the same patient receives different pseudonyms in different contexts [1]. Since EUPID stores the relationships between the pseudonyms, it can- provided the availability of necessary patient consents - facilitate linkage of identities and, subsequently, of data from different contexts at a later stage (privacy preserving record linkage - PPRL). EUPID does not store unencrypted versions of identifying variables like names or the dates of birth. Besides general aspects of the GDPR, the following requirements concerning personal rights were verified: Right on Rectification, Erasure, to be forgotten, Restriction of Processing, Portability and Objection.

**Results**

The GDPR emphasises pseudonymisation as a crucial measure for achieving data protection through technology and to diminish the risk for a personal data breach. Therefore, EUPID supports third-party systems to achieve GDPR-compliance. EUPID Services follow the principles of Privacy by Design (data minimisation, pseudonymisation, encoding/hashing, encryption, and key sharing restrictions including Trusted Third Parties) and Privacy by Default (minimisation of personal data processing, data encoding before transmission) and EUPID Services feature dedicated measures to fulfil the personal rights defined by the GDPR.

**Conclusion**

In conclusion, the EUPID Services are compliant with the applicable terms of the GDPR concerning the treatment of patient identification data and their registration as well as pseudonymisation. Future extensions will provide more advanced functionalities, in particular patient self-management and consent management.

**References**

1. Nitzlnader M, Schreier G. Patient identity management for secondary use of biomedical research data in a distributed computing environment. Stud Health Technol Inform, 2014; vol. 198, 211-8.

**Fig. 1 (abstract P32).**
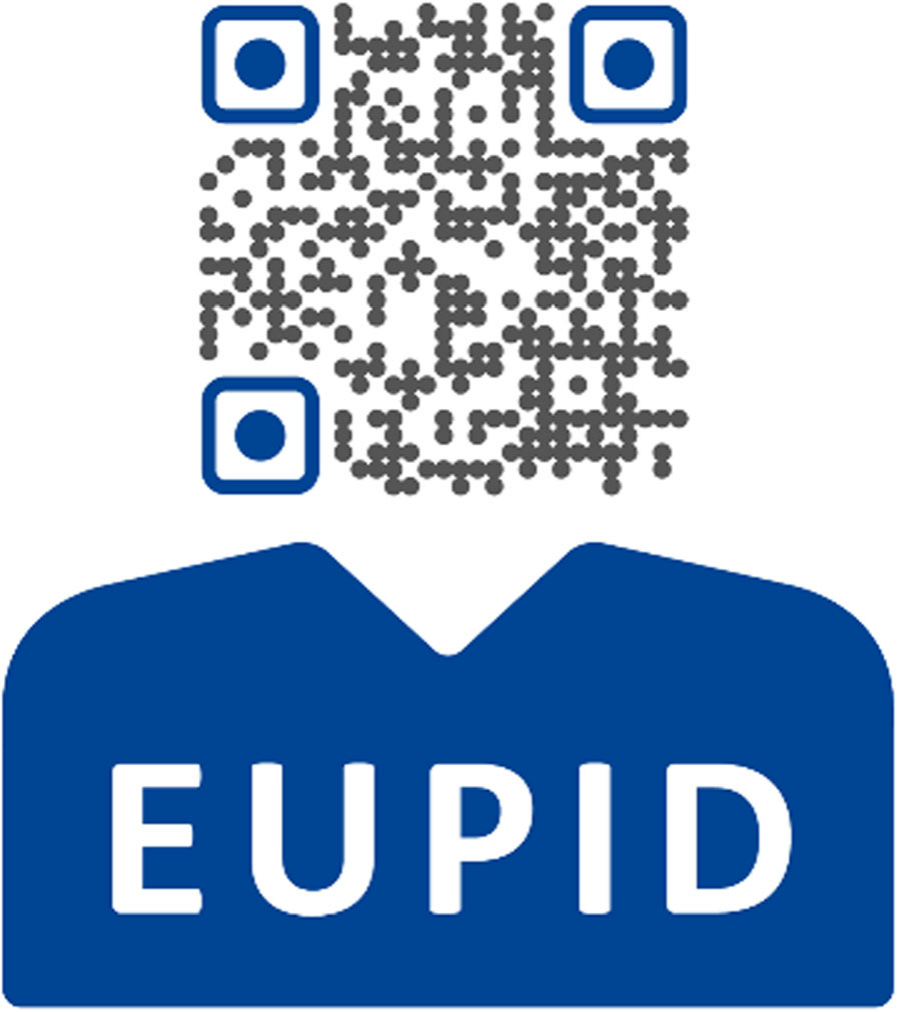
In order to easily recognize patient registration via EUPID Services, a dedicated logo has been created which contains a QR code that leads to the corresponding website: https://eupid.eu

### P33 Rare Diseases Registry in Basque Country: first steps

#### Luis M. Oregi Lizarralde, Luis J. Echevarría González-de-Garibay

##### Health Department, Basque Country Government, Vitoria-Gasteiz, Basque Country, 01010, Spain

###### **Correspondence:** Luis M. Oregi Lizarralde

The Basque Country of Spain has about 2.2 million inhabitants. Recently (2015) its Health Department established a Rare Diseases Registry (RER-CAE), as a part of its strategy on Rare Diseases (RD). This strategy also establishes specific RD committees in the main hospitals to help information dissemination and collaborative network.

The registry relies mainly on direct notification by clinicians. Hence, Basque Public Health Service (BPHS) has introduced an application form, whereby the specialists inform about the clinical aspects of diagnosed cases. This form, besides notifying the case to the registry, allows lighting a beacon in the system to let all clinicians know about the patients affected by rare diseases. The RER-CAE, along with this clinical information, collects personal data from BPHS databases, and disease-specific data gathered from Orphanet.

To encourage the participation of clinicians, and to ensure completeness, the cases which could refer to a group of about three hundred RD are systematically retrieved from BPHS databases. After a first screening, the RD potential cases are returned to clinicians, asking them to confirm RD real cases by filling out the application form.

Nowadays there are 3,105 cases registered, belonging to 3,090 patients, and standing for 479 different RD (Orpha codes). 86.4% reach “disease” granularity level and 13.6% belong to “group of diseases” granularity level.

The ten most prevalent diseases account for the 31% of the cases, among them: Neurofibromatosis type 1, Retinitis pigmentosa, Hereditary haemorrhagic telangiectasia, Autoimmune hepatitis or Idiopathic pulmonary fibrosis. Conversely, for now, 228 diseases have only one case registered, some of them very rare diseases, such as: Distal myopathy with anterior tibial onset, Hemorrhagic disease due to alpha-1-antitrypsin Pittsburgh mutation, X-linked intellectual disability-hypotonia-movement disorder syndrome, or Pierpont syndrome.

Using Orphanet’s linearity system for grouping the registered diseases, Developmental anomalies during embryogenesis (22.6%), Neurologic (17.8%), Systemic and rheumatological (8.5%), Inborn errors of metabolism (8.1%) and Endocrine (4.4%) are the most prevalent groups of diseases.

The participation of the different clinical services is highly variable, both between and within hospitals, being the more involved ones Internal Medicine (15% of registered cases), Genetics (14%), Digestive (11%), Pediatrics (10%) and Neurology (8%).

All these figures should be carefully considered, as the data are still too scarce to jump to highly significant conclusions. Incoming challenges include: to encourage clinicians’ implication; to return them registry-generated information; and to improve relationships with other registries, to both national and international scope, aiming to share information.

### P34 PID Genius: A Mobile Application by Patients for Patients Personal Assistant for Patients with a Primary Immunodeficiency (PID)

#### Martine Pergent^1^, Leire Solis^2^, Johan Prevot^3^, Saara Kiema^4^

##### ^1^IPOPI, Vice President, Estoril, Portugal; ^2^IPOPI, Health Policy and Advocacy Manager, Estoril, Portugal; ^3^IPOPI, Executive Director, Estoril, Portugal; ^4^IPOPI, NMO Programmes Officer, Estoril, Portugal

**Background**

PIDs are a group of 350 different rare genetic diseases occurring when components of the immune system do not work properly. Once diagnosed, PID patients need lifelong personalised treatment to lead as normal lives as possible. Treatments can be heterogeneous, such as immunoglobulin (IG) replacement therapy, anti-infectious prophylaxis, growth factors, anti-fungal medications etc.

PIDs being chronic diseases, adherence to treatment and medical follow-up are important. During regular visits to specialist(s) patients can report their tolerability to treatments. Keeping records of treatment history requires a lot of paperwork, especially for patients receiving IG therapy which, as human plasma derived medicinal products require rigorous compliance to reporting measures. This documentation is heavy to maintain and difficult to manage.

**Project leader**

IPOPI, International Patient Organisation for Primary Immunodeficiencies, launched the first mobile application developed by a patient organisation for PID patients for worldwide use.

**Objectives**

PIDGenius assists patients in:

- coping with their condition and treatment
- easing the discussion with specialists
- keeping track of key data and documents

**PIDGenius: different services**

- A profile with a summary of the patient and condition (Fig. 1)
- Health diary (Fig. 2) where patients can record their:
  - treatments
  - different parameters (physical, infections, mood, absences)
  - vaccines
  - appointments
- Description of treatments patient is on (Fig. 3)
- Dashboards displaying stored information, facilitating discussions with specialists (Fig. 4)
- Documents section, where patients have them at hand
- Emergency card summarizing information about the patient, condition and contacts.

**Method**

IPOPI set up two working panels, one of patients and one of physicians, to release an app based on patients’ needs and physicians’ views. Both panels were composed of people from different world regions to address items comprehensively, regardless of the country where the patient lives.

**Technical and legal issues**

PIDGenius complies with European regulation, one of the most rigorous legislations on personal data protection worldwide. PID Genius works on Android and iOS systems. Data are accessible on patients’ device on and offline, and stored on Azur cloud by Microsoft, offering the highest level of security to avoid any breach in patient privacy. The cloud is based in European Union. Only the patient can access personal data.

**Next steps**

The app is registered worldwide. The language of the app is English and will be translated into additional languages within the forthcoming months.

**Acknowledgement**

The development of this app was made possible thanks to the support of KEDRION BIOPHARMA

**Fig. 1 (abstract P34).**
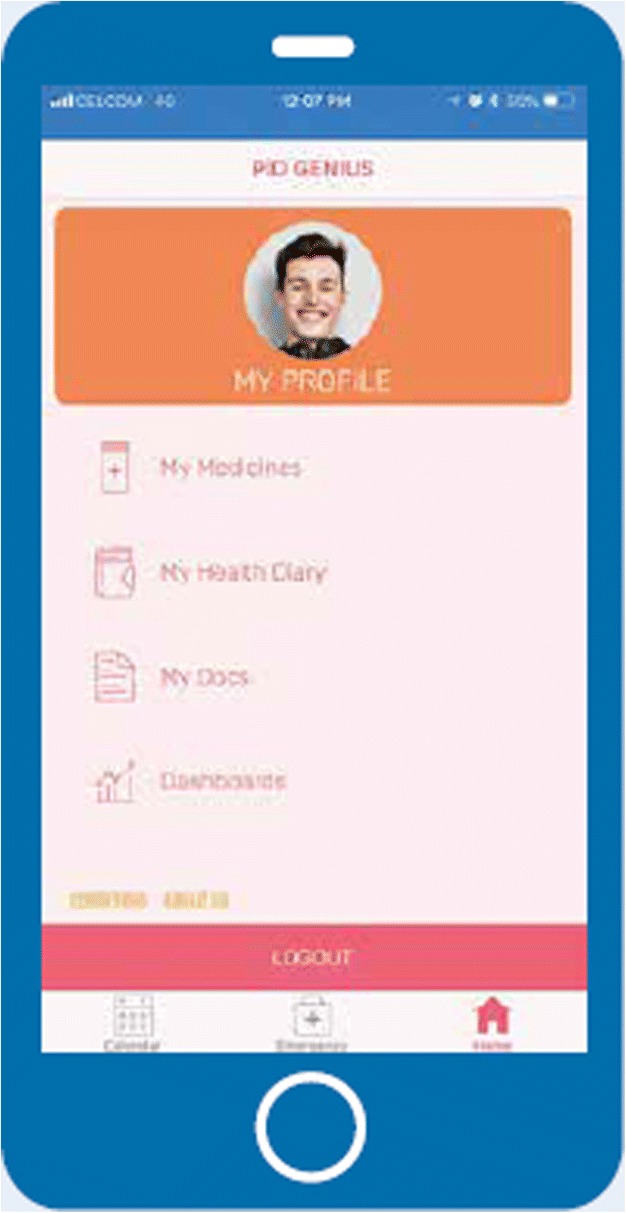
Profile

**Fig. 2 (abstract P34).**
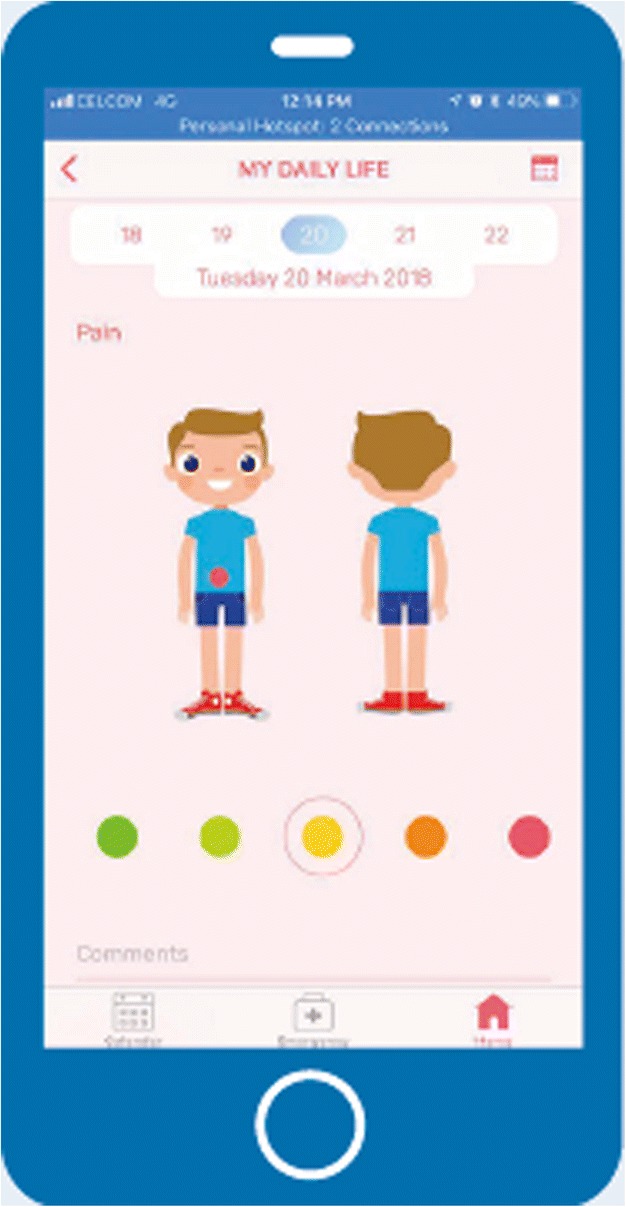
Health diary

**Fig. 3 (abstract P34).**
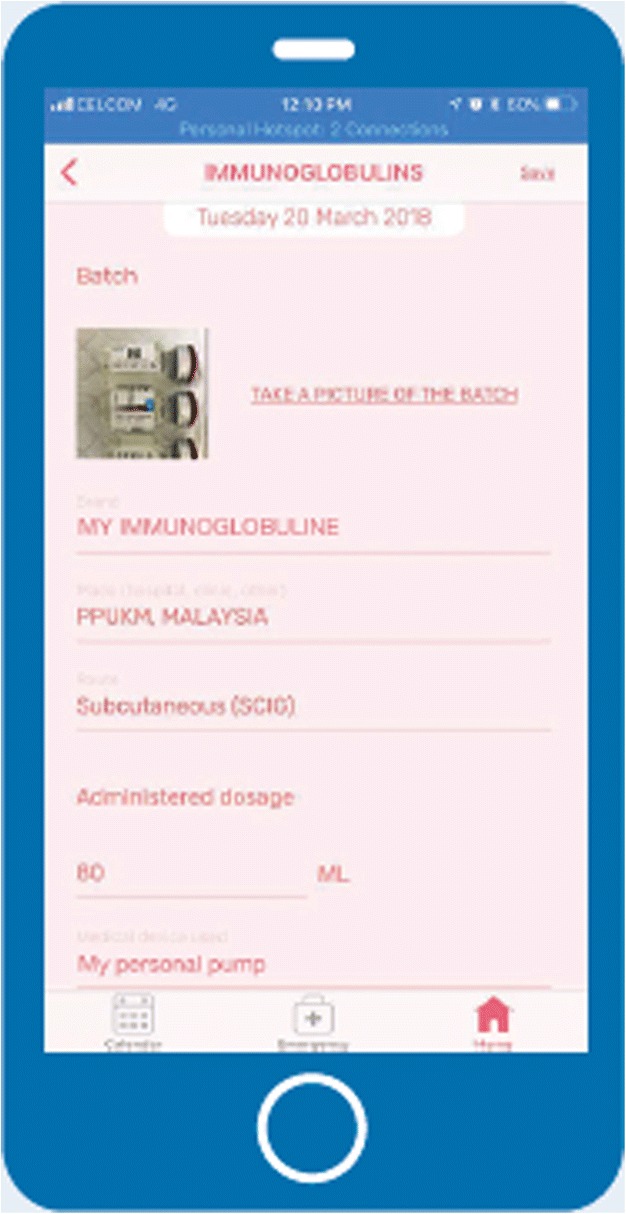
Treatments

**Fig. 4 (abstract P34).**
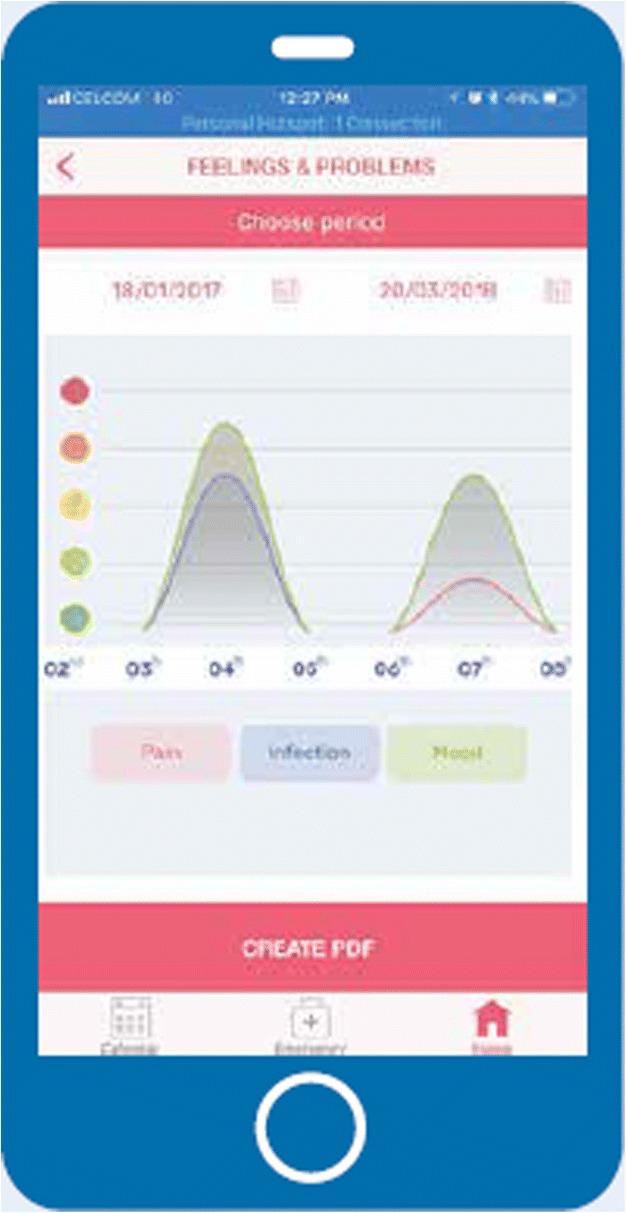
Dashboards

### P35 Registry of Lipodystrophy based on the OSSE-Framework

#### Jannik Schaaf^1^, Holger Storf^1^, Dennis Kadioglu^1^, Julia von Schnurbein^2^, Giovanni Cecarini^3^, Marie-Christine Vantyghem^4^, Camille Vatier^5^, Gabriele Nagel^6^, David Araujo-Vilar^7^, Martin Wabitsch^2^

##### ^1^Medical Informatics Group, University Hospital Frankfurt, Theodor-Stern Kai 7, 60590, Frankfurt, Germany; ^2^Center for Rare Endocrine Diseases, Department of Pediatrics and Adolescent Medicine – Division of Pediatric Endocrinology and Diabetology, University Medical Center Ulm, Ulm, Germany; ^3^Obesity and Lipodystrophy Center at the Endocrine Unit, University Hospital of Pisa, Pisa, Italy; ^4^CHU Lille, Endocrinology, diabetology and metabolism, Univ Lille, Lille, France; ^5^Sorbonne Université, INSERM UMR-S938 Saint-Antoine Research Center, and AP-HP, Hôpital Saint-Antoine, Department of Endocrinology, Paris, France; ^6^Institute of Epidemiology and Medical Biometry University Ulm, Ulm, Germany; ^7^Department of Medicine, University of Santiago de Compostela, Santiago de Compostela, Spain

###### **Correspondence:** Jannik Schaaf (schaaf@med.uni-frankfurt.de)

**Background**

The European Consortium of Lipodystrophy (ECLip) has set up a registry for patients with lipodystrophy using the OSSE (www.osse-register.de/en) open source software. The consortium consists of an association of European experts in the field of lipodystrophy. As lipodystrophy is an extremely rare disease divided into even rarer subtypes, research in this area is extremely difficult and international cooperation is essential [1].

The technical development and configuration of the registry was carried out by the Medical Informatics Group at the University Hospital Frankfurt supported by the IT administration team of the Institute of Epidemiology and Medical Biometry of the University Ulm.

**Methods**

The Open Source Software OSSE (Open Source Registry System for Rare Diseases in the EU) provides a software to set up a rare disease specific registry [2]. The fundamental goal of OSSE is to provide the possibility to create and establish a patient registry without extensive IT knowledge. The platform is web based and designed to comply to data protection requirements and preserve data sovereignty.

Before running the registry, the data structure as well as the case report forms (CRF) have been defined. The users of the different sites of the ECLip Group were involved early in this process to define a harmonized dataset for the registry. In addition, the group developed a patient consent form and a data protection concept. After setting up the server of the registry, provided by the University Hospital Ulm, the dataset was defined in the central metadata repository (MDR). The use of this central MDR allows to specify the data elements collected in the registry [3, 4]. The goal of this approach is to have consistent records with detailed descriptions and units of each items. Thus, it is possible to improve the quality of the documentation.

**Results**

The final result is a patient registry for the collection of data on patients with lipodystrophy, based on the software system OSSE. In the process, more than 400 collected data elements were created in finally 35 different base- or longitudinal CRF. Moreover, the productive system was started and the first patient data was entered after a positive ethic vote of the individual locations.

**Discussion**

By providing a platform for the networking of different experts for lipodystrophy from different countries and medical disciplines, it will become possible to compare diagnosis of and care for affected patients across Europe.

**References**

1. Brown RJ, Araujo-Vilar D, Cheung PT, Dunger D, Garg A, Jack M, et al. The Diagnosis and Management of Lipodystrophy Syndromes: A Multi-Society Practice Guideline. J Clin Endocrinol Metab*.* 2016; 101(12): 4500-4511.

2. Muscholl M, Lablans M, Wagner TOF, Ückert F. OSSE – open source registry software solution. Orphanet Journal of Rare Diseases. 2014.

3. Michalik C, Ngouongo S, Stausberg J [Internet]. Beschreibung der IT-Anforderungen an Kohorten und Register. Technologie- und Methodenplattform für die vernetzte medizinische Forschung (TMF). 2015 [cited 20 June 2018]. Available from: https://www.toolpool-gesundheitsforschung.de/produkte/it-anforderungen-kohorten-register

4. Drepper J [Internet]. Das Nationale Metadata Repository – Standardisierte Datenelemente für die patientenorientierte Forschung. Technologie- und Methodenplattform für die vernetzte medizinische Forschung(TMF). 2013 [cited 20 June 2018]. Available from: http://www.tmf-ev.de/DesktopModules/Bring2mind/DMX/Download.aspx?Method=attachment&Command=Core_Download&EntryId=22277&PortalId=0

### P36 3 Years se-atlas - Mapping of Health Care Providers and Support Groups for People with Rare Diseases

#### Johanna Schaefer^1^, Niels Tegtbauer^1^, Thomas O F Wagner^2^, Holger Storf^1^

##### ^1^Medical Informatics Group, University Hospital Frankfurt, 60590 Frankfurt am Main, Germany; ^2^Frankfurt Reference Centre for Rare Diseases, University Hospital Frankfurt, 60590 Frankfurt am Main, Germany

###### **Correspondence:** Johanna Schaefer (j.schaefer@med.uni-frankfurt.de)

**Background**

Finding specialized health care providers is often a huge challenge for people with Rare Diseases (RD) and their relatives [1]. Helping to tackle this problem in Germany, the project se-atlas – Mapping of Health Care Providers for People with Rare Diseases started in 2013. The Federal Ministry of Health in Germany initially funded the project, being part of the National Plan of Action for People with Rare Diseases [2]. The objective of se-atlas is to present health care providers and support groups related to RD in Germany in an interactive map view as well as in form of a list [3]. Since its launch in 2015 the platform is online available at www.se-atlas.de.

**Methods**

After developing and testing the platform, the database of se-atlas has still to be filled with specialized health care providers and support groups for RD. To achieve a high-quality-based dataset, an editor team took up the work, reviews new entries and updates the database periodically. In the course of the project period, different data sources were migrated. Besides data from disease related expert associations, German members of the European Reference Networks and data from the project partner Orphanet Germany, many data were transmitted from support groups to se-atlas. To underline the importance of support groups and their expertise for RD, they can additionally affirm health care providers, which are specialized for their disease. Furthermore, experts for RD registered by themselves and proofed by the editor team are part of the current database.

**Results and Outlook**

Since the launch of se-atlas in March 2015, the database is growing steadily. Today the platform includes 1574 special consultations or special ambulances, more than 850 subordinated health care facilities and 198 parent facilities as well as 366 support groups for RD, including the members of Alliance of Chronic Rare Diseases (ACHSE e.V.). Besides the growing database, the number of visitors and external links are rising continuously which demonstrates the relevance of se-atlas and results in a better SEO-ranking at Google and other search engines.

Even after 3 years se-atlas.de the data is not completed. Therefore, the overall goal is continuing filling the database in particular with the help of active support groups and further ensuring the quality of the data in the long term.

**Project Partner**

Treatment and Research Centre for Rare Diseases Tuebingen, Germany; Frankfurt Reference Center for Rare Diseases, Germany; Orphanet Germany; Alliance of Chronic Rare Diseases e.V., Germany

**References**

1. Eidt D, Frank M, Reimann A, Wagner TOF, Mittendorf T, von der Schulenburg J-M. Maßnahmen zur Verbesserung der gesundheitlichen Situation von Menschen mit Seltenen Erkrankungen in Deutschland. Forschungsbericht Studie im Auftrag des Bundesministeriums für Gesundheit. 2009; [https://www.bundesgesundheitsministerium.de/fileadmin/Dateien/5_Publikationen/Praevention/Berichte/110516_Forschungsbericht_Seltene_Krankheiten.pdf] Accessed 5 March 2018.

2. Geschäftsstelle des Nationalen Aktionsbündnisses für Menschen mit Seltenen Erkrankungen. National Plan of Action for People with Rare Diseases. Action Fields, Recommendations, Proposed Actions.2013; [http://www.namse.de/images/stories/Dokumente/Aktionsplan/national%20plan%20of%20action.pdf] Accessed 5 March 2018.

3. Haase J, Wagner TOF, Storf H. se-atlas – Versorgungsatlas für Menschen mit Seltenen Erkrankungen. Unterstützung bei der Recherche nach Versorgungseinrichtungen und Selbsthilfeorganisationen. Bundesgesundheitsblatt Gesundheitsforschung Gesundheitsschutz. 2017; 60:503-509.

### P37 German National Plan of Action for People with Rare Diseases: further developments

#### Miriam Schlangen^1^, Katharina Heuing^1^

##### ^1^Coordination Office of the National Action League for People with Rare Diseases, c/o Mukoviszidose Institut gGmbH, Bonn, Germany

###### **Correspondence:** Miriam Schlangen (mschlangen@namse.de)

**Objective**

Four years after its publication, the implementation of the National Action Plan (NAP) is still forced by the members of the National Action League for People with Rare Diseases (NAMSE). In the following we will outline a general overview of what has been successfully finished and the next steps.

**Method**

Since the publication of the NAP, the state of progress in implementing its action and projects has been monitored regularly on the basis of information from the responsible NAMSE partners. With the help of this information, measures can be divided into different degrees of implementation. To ensure the long-term implementation and continual further development of the Action Plan, a sustainable organisational structure for NAMSE has to be developed.

**Results**

Directive 2011/24EU on the application of patients’ rights in cross-border healthcare envisages the implementation of European Reference Networks (ERN). These are networks connecting national health care providers and national centres of expertise of highly specialised healthcare, for the purpose of improving access to diagnosis, treatment and the provision of high-quality healthcare for patients. On 1st March 2017, 24 thematic European Reference Networks began to work, gathering together over 900 highly specialised healthcare units from 26 countries. 120 German healthcare units out of 58 hospitals are involved. A main focus of the German NAP is still the establishment of the centres’ scheme for rare diseases with centres of expertise. Therefore, a consensus was reached on the criteria catalogues for type A (reference) centres and type B (expertise) centres by NAMSE.

The evaluation of the criteria catalogues is the main task of NAMSE. Therefor a new structure of the National Action League has to be build. Moreover, in several workshops all agreed, that NAMSE should stay the national council responsible for coordinating and publishing the common efforts.

**Conclusion**

Four years after the publication of the National Action Plan most of the proposed measures have been implemented. Some of the measures are due to the establishment of the centres’ scheme for rare diseases. Main goal is currently to make NAMSE fit for further actions by continuing the National Action League on a sustainable basis. Details are currently under discussion.

### P38 Measuring the effects of a case management approach on the quality of life of rare and complex disease patients in Salaj, Romania: a pilot randomised control trial of efficacy

#### Juliet Tschank, Katharina Handler, Stefanie Konzett-Smoliner

##### Work and equal opportunities, Centre for Social Innovation, Vienna, 1150, Austria

###### **Correspondence:** Juliet Tschank (tschank@zsi.at)

**Trial design**

The efficacy of the INNOVCare pilot is measured based on a basic two-condition repeated-measures design [1] or rotation design [2] which ensures that all participants receive the intervention; just at different times [3].

**Methods**

Participants are rare and complex disease patients and their families living in the county of Salaj in Romania. All 60 beneficiaries from the NoRo Centre implementing the intervention were automatically eligible. To add an element of external validity [4], further 60 individuals were randomly sampled from the rare disease registry of the county using proportional stratified sampling [5].

All 120 participants were randomly allocated into two cohorts using randomised block design [4]. The first received the intervention during the first nine months and the second, during the following nine months of the pilot (Fig. 1).

**Intervention**

The case management services offered to the participants aims at relieving their burden of care management by linking health services to employment, social and support services.

**Objectives**

The overarching goal of the INNOVCare pilot is to improve quality of life of the participants. Direct objectives include improvement in:

1. Self-management of care
2. Communication skills
3. Disease-related peer-to-peer learning
4. Understanding and acceptance in community
5. Coordination of care among stakeholders and knowledge about:
6. Disease
7. Patient rights
8. Services

Quality of life is measured using generic instruments while specific objectives are measured using tailored questions. The resulting questionnaires for patients and their families were completed at three points in time (Fig. 1).

**Results**

Preliminary results (from the first two measurements) show that the intervention has no significant impact on quality of life, but show significant improvement in six of the eight specific objectives of the intervention. The two exceptions are: increasing “acceptance in the community” and “peer-to-peer learning” (Fig. 2).

**Conclusion**

So far, the intervention can be considered successful in terms of its social impact e.g. patients are more empowered, informed and have higher self-confidence. Although there were no measurable effects regarding quality of life, it should be considered that quality of life: Can only be affected indirectly, is a complex phenomenon and requires time to improve considerably and is additionally affected by external factors e.g. disease and degree of disability.

**Acknowledgements**

This work is part of the INNOVCare project (www.innovcare.eu), funded by the European Union (Call for Proposals VP/2014/008; EaSI PROGRESS, DG Employment, Social Affairs and Inclusion).

**References**

1. Field, A. & Hole, G., 2003. *How to Design and Report Experiments.* Wiltshire: SAGE Publications Ltd.

2. Glennerster, R. & Takavarasha, K., 2013. *Running randomized evaluations: a practical guide.* New Jersey: Princeton University Press.

3. Tschank, J., Handler, K. & Gruber, N, 2017. *Methodology report: The effects of a case management approach on the quality of life of rare and complex disease patients in Salaj, Romania: a randomised control of efficacy*. [Online]. Available at: https://innovcare.eu/wp-content/uploads/2017/07/INNOVCare_WP7_Methodology-report_website.pdf

4. Verma, J. P., 2016. *Repeated measures design for empirical researchers.* New Jersey: Wiley.

5. Kumar, R., 2005. *Research methodology: A step-by-step guide for beginners.* Second ed. Malaysia: Sage Publications, Inc.

**Fig. 1 (abstract P38).**
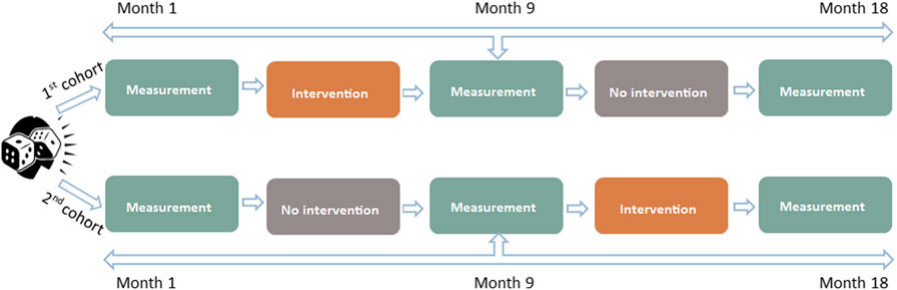
Trial design

**Fig. 2 (abstract P38).**
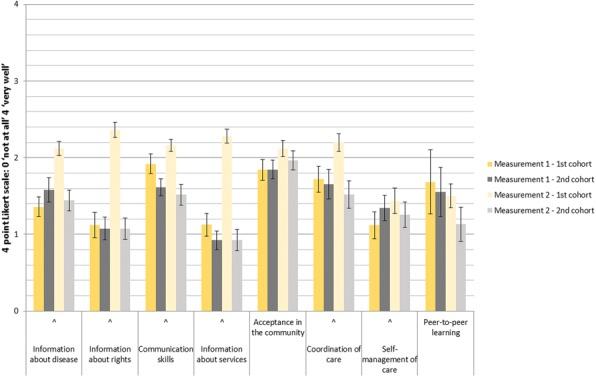
Preliminary results

### P39 The TREAT-NMD Advisory Committee for Therapeutics: A multi-disciplinary expert approach to drug development advice

#### Cathy Turner^1^, Volker Straub^1^, Kathryn Wagner^2^

##### ^1^John Walton Muscular Dystrophy Research Centre, Newcastle University, Newcastle upon Tyne, NE17RU, UK; ^2^The Kennedy Krieger Institute, Johns Hopkins School of Medicine, Baltimore, MD21205, USA

###### **Correspondence:** Cathy Turner (catherine.turner@ncl.ac.uk)

**Background**

The TREAT-NMD Advisory Committee for Therapeutics (TACT) was established to provide independent and objective guidance on the preclinical and development pathway of potential therapies for rare neuromuscular diseases [1].

**Some Challenges in Therapy Development for Rare Neuromuscular Disease**

For a number of reasons, most clinical trials in rare disease fail (Fig. 1).

- Fewer than 90% of compounds that enter Phase 1 clinical trials result in a marketed drug
- Drug development is expensive. Where there are few patients, it is even more expensive
- Patient numbers in rare disease are small and strict inclusion/exclusion criteria alongside competing trials mean recruitment targets for trials need to be low
- For slowly progressing conditions, trials are often longer than average which increases the burden on patients
- Lack of natural history data means a placebo arm is usually required. In long trials for degenerative muscle diseases - often involving children - this is a high burden on patients
- Agreement on clinically meaningful outcome measures, approved by regulators, is difficult
- Groups planning trials in this field may be new to the disease and have little understanding of the condition
- A lack of regulatory guidance early on can make subsequent registration and approval more difficult

**Part of the solution: The TREAT-NMD Advisory Committee for Therapeutics (TACT)**

- Established to provide independent and objective guidance on the pre-clinical and development pathway of potential therapies for all rare neuromuscular diseases. The majority of applications have been in Duchenne muscular Dystrophy (DMD) although a wide range of disease areas have been covered (Fig. 2)
- A multidisciplinary body of drug development experts that includes patients and/or their advocates
- Works closely with patient organisations in multiple regions to ensure the process meets the needs of the neuromuscular community as a whole
- A bespoke expert panel of world-leading experts from all areas of drug development discuss each application together and with the applicants before formulating comprehensive and confidential advice
- Generates recommendations that have greatly helped investigators in evaluating their potential compounds and in considering their development program in a methodical fashion with clear go/no-go decisions
- So far 46 applications from industry and academics have been received and reviewed over 8 years

**How TACT works**

1. Applicants working on the development of a therapy in NMD outline their proposals – these can be at a pre-clinical stage right through to phase III or even post-marketing
2. Under strict confidentiality agreements and checking for conflicts, a panel of reviewers (10-15) is convened every 6 months in response to the particular needs and questions of applications
3. A 6-week online review between the experts is followed by a face-to-face meeting and discussion with the applicant
4. A comprehensive and confidential advisory report is sent to the applicant after the meeting
5. TACT does not recommend projects to be funded but a review is often used to help support funding applications – indeed, funders often refer applicants to TACT

**Acknowledgements**

Submitted for and on behalf of the TACT Core Committee: http://www.treat-nmd.eu/resources/tact/committee-members/

Generous funding for TACT has been received from: EU FP6, CNMC (via DoD grant), PPMD, Cure Duchenne, MDUK, MDA, Joining Jack, Duchenne UK, Duchenne Ireland, Myotubular Trust, Duchenne Now, Duchenne Children’s Trust, SMA Europe

**References**

1. Heslop E, Csimma C, Straub V, McCall J, Nagaraju K. et al, The TREAT-NMD advisory committee for therapeutics (TACT): an innovative de-risking model to foster orphan drug development. Orphanet Journal of rare Diseases. 2015; 10:49

**Fig. 1 (abstract P39).**
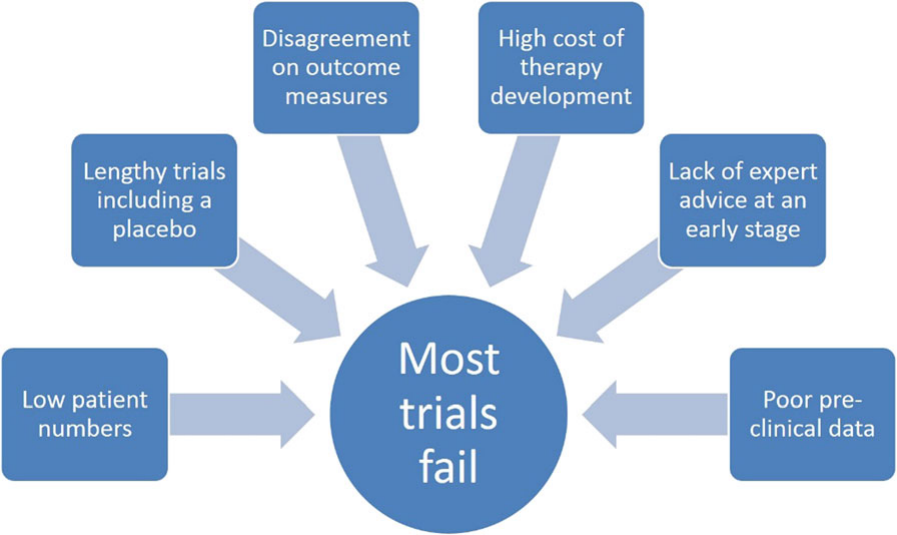
The majority of clinical trials in rare disease do not result in the delivery of a therapy

**Fig. 2 (abstract P39).**
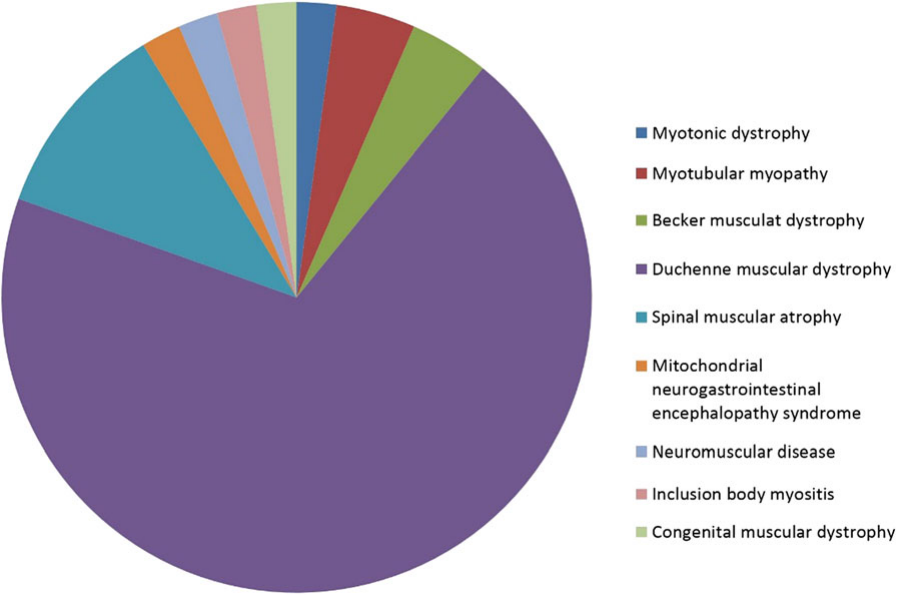
TACT applications by disease area

### P40 Bulgarian Acromegaly Database clinical studies

#### Silvia Vandeva^1^, Emil Natchev^1^, Atanaska Elenkova^1^, Dimitar Tcharaktchiev^1^, Krassimir Kalinov^2^, Sabina Zacharieva^1^

##### ^1^Clinical Center of Endocrinology and Gerontology, Medical University – Sofia, Sofia 1431, Bulgaria; ^2^ Department of Informatics, New Bulgarian University, Sofia 1618, Bulgaria

###### **Correspondence:** Silvia Vandeva

**Background**

In 2009 the Bulgarian Acromegaly Database was created with the collaboration of all university endocrinology centres in the country, covering practically all patients with acromegaly. Using the database, we conducted several clinical studies, evaluating some epidemiological characteristics, effect of different treatment modalities and quality of life in Bulgarian acromegaly patients.

**Methods**

Using the database, we evaluated incidence and prevalence of acromegaly in our country [1]. In a collaborative study with Campania we compared mortality rates between 407 Bulgarian and 212 Italian patients for the period 1999-2008 [2]. In a retrospective manner we evaluated the biochemical outcome in 534 patients over a 30-year period of time (1980-2012) [3]. We assessed quality of life (QoL) using the Acromegaly Quality of Life Questionnaire (AcroQoL) in 212 patients over a six-year period of time in a cross-sectional manner, and 70 patients of them were followed-up prospectively [4].

**Results**

Based on 792 patients we found a maximal incidence in the 80s – 2.9 cases/million/year, prevalence of 48.4 cases/million, and 1.7:1 female/male ratio. In the mortality study the Bulgarian patients had decreased life expectancy compared to the general population, while the Italian cohort had normal life expectancy (Table 1).

The main difference between both cohorts was higher percentage of disease control in the Italian patients 50.1% vs. 39.1%, p<0.05, due to the use of somatostatin analogues (SSAs) and Pegvisomant that were lacking in Bulgaria during the study period. Table 2 shows the effect of different treatment approaches on normalization of both GH and IGF-1.

In regards to the QoL, in the cross-sectional group female gender, older age, lack of disease control, prior radiotherapy were independently related to worse scores of different scales of AcroQoL. In the prospective group achievement of remission, SSA treatment and lack of hypopituitarism predicted improvement of the scores of some of the scales.

**Conclusions**

Suboptimal treatment of acromegaly is related to higher mortality rates. Dopamine agonist treatment is effective in approximately one-fifth of the patients. Quality of life is a multifactorial issue that needs further investigation.

**References**

1. Vandeva S, Orbetzova M, Tcharaktchiev D, Kirilov G, Elenkova A, Atanasova I, et al. Acromegaly in Bulgaria - Epidemiological Characteristics Derived from the National Acromegaly Database. Endocrinologia. 2010;XV:142-50.

2. Colao A, Vandeva S, Pivonello R, Grasso LF, Nachev E, Auriemma RS, et al. Could different treatment approaches in acromegaly influence life expectancy? A comparative study between Bulgaria and Campania (Italy). Eur J Endocrinol. 2014;171:263-73.

3. Vandeva S, Elenkova A, Natchev E, Kirilov G, Tcharaktchiev D, Yaneva M, et al. Treatment outcome results from the Bulgarian Acromegaly Database: adjuvant dopamine agonist therapy is efficient in less than one fifth of non-irradiated patients. Experimental and clinical endocrinology & diabetes : official journal, German Society of Endocrinology [and] German Diabetes Association. 2015;123:66-71.

4. Vandeva S, Yaneva M, Natchev E, Elenkova A, Kalinov K, Zacharieva S. Disease control and treatment modalities have impact on quality of life in acromegaly evaluated by Acromegaly Quality of Life (AcroQoL) Questionnaire. Endocrine. 2015;49:774-82.

**Table 1 (abstract P40).** Mortality ratios in Bulgaria and Campania.

Mortality	SMR	95% CI	P value
All-cause, BG cohort	2.0	1.54-2.47	<0.05
All-cause, BG cohort in remission	1.25	0.69-1.89	NS
Cerebrovascular, irradiated, BG cohort	7.15	2.92-11.37	<0.05
Cerebrovascular non-irradiated, BG cohort	2.28	0.87-3.69	NS
All-cause, IT cohort	0.66	0.27-1.36	NS

*SMR* standardized mortality ratio, *CI* confidence interval, *BG* Bulgarian, *IT* Italian, *NS* non-significant

**Table 2 (abstract P40).** Remission rates in respect to different treatment modalities.

Mode of treatment	Remission rates (% of patients)	Total number of patients
Overall	51.4	534
Overall after introduction of SSA	70.3	219
Surgery	28.8	486
Adjuvant CAB without prior Ro	18.2	70
Adjuvant SSA	38.6	70
Conventional radiotherapy	34.6	159

*CAB* Cabergoline, *Ro* radiotherapy, *SSA* somatostatin analogs

### P41 Sharing good practices of the daily life with Gaucher disease

#### Irena Žnidar, Thomas Biegler, Davor Duboka, Gil Faran, Jasenka Wagner

##### European Gaucher Alliance, 8 Silver Street, Dursley, Gloucestershire, GL11 4ND, UK

###### **Correspondence:** Irena Žnidar (irena@eurogaucher.org)

Gaucher organizations worldwide have a common voice in the European Gaucher Alliance (EGA), which is the umbrella organization of 47 national organizations. Treatment of Gaucher patients varies in the EGA member countries. There are many examples of good practice in individual countries.

The examples of good practice were collected in 2015 through a questionnaire asking about the routine monitoring tests, enzyme replacement therapy (e.g. home treatment, equipment for intravenous infusion), substrate reduction therapy and the other aspects of the daily life of Gaucher patients (e.g. travelling, privileges, meetings). 131 patients from 22 countries responded to the questionnaire. A reader-friendly brochure has been prepared which will be shared with the Gaucher community intending to help improve daily life with Gaucher disease.

Some examples of good practice:

- All Gaucher patients from my country travel on the same day to the same hospital where we all undertake monitoring tests. This also proves a good opportunity to meet each other.
- For children, the regular check-ups are done during school holidays, so they are not absent from school.
- I meet other Gaucher patients during enzyme replacement therapy at the hospital.
- The nurse comes to every place in the country regardless of where I am staying, even if I am away from home.
- I collect the drug at the local pharmacist in my hometown just before having enzyme replacement therapy at home. I transport the drug in a cool bag and store it in a kitchen refrigerator (I check that the temperature is 2-8 °C) until the infusion is administered at home.
- When I am home alone, I use a “Y-system”, which means I connect both bottles (one with the drug and the other with saline for flushing) to the system. When the first bottle is empty, I only open the flow of the second bottle. When I don’t use a “Y-system”, I ask somebody to change the bottles for me, as this is quite difficult to manage by myself, as the metal needle in the vein should not move too much.
- A telescopic intravenous stand, designed for home treatment, is very useful and it does not remind you on the hospital equipment. The weight of this stand is 2 kg and when folded it does not take much space and it is easy to carry.
- With several weeks of vacation, I take my infusion two weeks on a row upfront and possibly after returning again two weeks on a row.
- Longer patient meetings (e.g. for two days) are better than one-day meetings, as the participants become more relaxed and open, especially when we discuss difficult or personal topics.

## Speaker presentations

### S1 The challenges and opportunities of crowdfunding rare disease research

#### Heather C. Etchevers

##### Aix-Marseille Univ, Marseille Medical Genetics (MMG), INSERM U1251, Marseille, France

In 2014, with the assistance of the Blackswan Foundation and its rare disease crowdfunding platform, RE(ACT) Community, I raised donations of over 33,000 euros from more than 80 individual backers for my academic research group. The stated goal of our project was to constitute a French national registry and biobank devoted to the rare birth defect known as the giant congenital melanocytic nevus. This resource is now a reality, though funding remains extremely limited to fully exploit its potential. Our experience was rich in lessons. What are measures of success in terms of greater knowledge about the subject of study, benefit to patients, or scientific crowdfunding in general? How satisfied are the people involved in conceiving of, promoting and finally getting to work on crowdfunded projects, and who are they? In this presentation, I also referred to two other recent examples of crowdfunded rare disease-related projects, the “Lil’BUBome” and the “Cure Black Bone Disease” projects, to pinpoint both intermediate and unmitigated successes. A common lesson learned was that scientific crowdfunding can be more of an exercise in scientific and rare disease communication exchange with the general public, than a useful investment of time from a fundraising perspective. Campaigns need to be entertaining or distracting, in order to retain the attention necessary to open wallets to support it. Another lesson was that it is certainly not a simple way of raising funds or predictably proportionate to the effort required. Anyone considering a new crowdfunded project needs to plan a calendar for their entire campaign many months before launch, having gathered a small team of motivated partners with defined roles concerning the actual project advancement, plus video shoots, webpage and social media updates, conceiving of stretch goals, and other classical management aspects – in addition to their day jobs, often as scientists. Success can be measured in terms of attaining modular and self-sufficient funding goal levels during the campaign, involving multiple people affected by the condition studied, obtaining further grants from more traditional sources, producing peer-reviewed publications or other tangibles based on results obtained, earning positive attention for the institutions in which such projects are taking place, and ultimately gaining and disseminating knowledge of direct benefit to the rare disease community.

### S2 Patient involvement in EUnetHTA rapid Relative Effectiveness Assessments

#### Sabine Ettinger^1^, Anne Willemsen^2^, Julia Mayer-Ferbas^1^, Ida-Kristin Ø. Elvsaas^3^, Ingvil Saeterdal^3^

##### ^1^Ludwig Boltzmann Institute for Health Technology Assessment, Garnisongasse 7/20, 1090 Vienna, Austria; ^2^National Health Care Institute, Eekholt 4, 112XH Diemen, Netherlands; ^3^The Norwegian Institute of Public Health, PO Box 222, Skøyen, 0213 Oslo, Norway

###### **Correspondence:** Sabine Ettinger (sabine.ettinger@hta.lbg.ac.at)

**Background**

There is consensus among experts that the involvement of patients and citizens in health technology assessment (HTA) processes is valuable since they can provide specific insights into aspects of their disease, health-related quality of life, available therapies and relevant outcomes. The European Network for HTA (EUnetHTA), funded by the European Commission, recognised the need for improved and standardised involvement processes in its HTAs (rapid relative effectiveness assessments).

**Methods**

We aimed at testing different methods for patient involvement in HTAs on pharmaceuticals and non-pharmaceutical technologies: Patient input template (open questions sent via Email to relevant patient organisations) (n= number of assessments: 1); scoping meetings with patients/patient representatives (n=2); semi-structured interviews (n=3) and focus groups (n=2) that are recorded, transcribed and analysed. European patient & consumer organisations provided input to the overview of preferred methods. The respective method chosen was dependent on the topic of the HTA and different circumstances (like timelines of HTA and burden on the patient) were considered.

**Results**

Patients were included in 2 of 3 published pharmaceutical HTAs by means of semi-structured interviews and in 3 of 6 published HTAs in non-pharmaceutical technologies by means of focus group, scoping meeting, and other approach (i.e. written feedback on scope - as tested out in the beginning). Three of 7 ongoing HTAs in non-pharmaceutical technologies included patients (focus groups, scoping meeting, semi-structured interviews, patient input template). Due to inability of identifying suitable patients or tight timelines it was not possible to include patients in all HTAs. Results gathered provided patients’ perspectives on health-related quality of life and informed outcome measures. Involvement of patients in the early stages of an HTA i.e. in defining the scope was shown most useful. General challenges identified were identification of participants representative for the patient group, patient involvement within given timelines and how to handle input from a limited number of patients. One of the pharmaceutical HTAs and none of the non-pharmaceutical technologies was dealing with a rare disease.

**Conclusions**

The different approaches proved useful for complementing HTA processes. However, further testing and evaluation of these approaches is needed to gain insights into the impact of patient involvement on European HTAs and to refine the methods for patient involvement. Currently, EUnetHTA is working to establish a procedure for topic identification, selection and prioritization where proposals from stakeholders i.e. input from patient organisations will play a role.

**Acknowledgements**

The authors would like to thank the EUnetHTA Task Group on Patient and Health Care Provider Involvement for their valuable contribution in coming up with preferred methods for patient involvement that can be further tested.

### S3 Quality of life in Health Technology Assessment: the Dutch experience

#### Pauline Evers^1^ Angeline Jansen^2^

##### ^1^Dutch federation of cancer patient organisations, Utrecht, The Netherlands; ^2^AA and PNH contact group, Amsterdam, The Netherlands

###### **Correspondence:** Pauline Evers (p.evers@nfk.nl)

In The Netherlands the Zorginsitituut (ZINL) advises the ministry of Health on inclusion of new therapies in the reimbursement. Two examples were presented on how Quality of life was considered during the evaluation.

**Eculizumab for treatment of Paroxysmal Nocturnal Hemoglobinuria (PNH)**

PNH is a rare disease where destruction of red blood cells is caused by a misfunctioning of the C5 (part of the immune system). This leads to anemia and clot formation in blood vessels and to severe fatigue and iron deficiency. The standard of care was frequent blood transfusion and the use of anti-clotting agents and, if a donor was available, stem cell transplantation. By binding to C5 eculizumab stops the breakdown of red blood cells. Cost: 360.000€ p/yr.

After registration in 2007 the product got conditional reimbursement and around 70/140 PNP patients are treated with eculizumab in the Nijmegen expert center.

During the re-evaluation (2016) ZINL concluded the product was marginally effective and certainly not cost-effective. The patient organization, in collaboration with the expert center, collected real world evidence data on Quality of life and participation in daily life activities. Results showed that the labor participation of patients who received eculizumab from diagnosis was almost equal to the general population as was their quality of life. Based on these results and after price negotiations by the ministry of health the product continues to be reimbursed.

The work of the patient organisation did pay of and real world data from a non-validated survey were taken into account.

**Mammaprint (MP) in breast cancer**

Patients with primary breast cancer can get adjuvant chemotherapy after surgery to prevent recurrence of the disease. The need for chemotherapy is determined based on clinical items (seize of the tumor, nr of lymph nodes involved, histology etc). The MP is a genetic test resulting in a more detailed picture. Around 23% of patients have a high risk clinical and a low risk MP so they have the option to refrain from toxic chemotherapy. The MINDACT study showed a 5-year disease free survival (DFS) of 94.5% based on clinical prediction and 92% based on MP.

Patients and physicians argued that the fact that 23% less patients received chemotherapy after applying MP (associated with side effects and Quality of life) outweighed the small deterioration in DFS.

The MP was assessed through EUnetHTA and the outcome was negative. To the opinion of patients and doctors QoL of life was insufficiently taken into account.

### S4 Integrated care – patient perspective Sweden

#### Beata Ferencz (beata.ferencz@sallsyntadiagnoser.se)

##### Rare Diseases Sweden, Stockholm, Sweden

Sweden is one of the few countries that has yet to adopt a national plan for Rare Diseases. Several independent agencies and authorities have acknowledged major shortcomings with coordinated care and continuity within the Swedish healthcare system. This impacts individuals with a rare disease extensively. Knowledge and research is scarce, diagnosis is delayed, there is a lack of coordination and access to treatment is unequal. See Fig. 1 for an illustration of the points of contact within a care system that a caregiver needs to coordinate for an individual with a rare disease.

Williams syndrome is caused by a spontaneous deletion of 26-28 genes on chromosome 7. Children with Williams syndrome have a complex mix of extraordinary gifts and challenges. They are often social, friendly and endearing and bring an immense joy and perspective to their families. Nonetheless, a set of challenges can be present including potentially life-threatening cardiovascular problems including anesthesia concerns, ADHD, general anxiety disorder and delays in motor and speech development. Children need early intervention and coordinated medical care to avoid potentially life-threatening situations. Despite the presence of extensive medical guidelines for Williams syndrome the care is still highly dependent on caregivers [1].

The Swedish habilitation system offers treatments to children and adults with disability to develop motor, language and communication skills. They also offer great programs in terms of education for parents and other community support services. While they meet many of the early intervention needs the resources are scarce and the access to habilitation is unequal. Currently efforts to coordinate care is limited in Sweden and there is a necessity to develop multidisciplinary teams/and or clinics. The Swedish Center for Rare Diseases have the potential to offer solutions and while they are promising it is necessary that they receive more resources to meet the needs of the rare disease community.

It is essential that we recognize rare diseases as a public health priority. While each diagnosis may be rare, there are approximately 400 000 individuals living with a rare disease in Sweden today. Yet, this is a group that essentially doesn’t exist in population studies or registries. In order to provide the necessary care for individuals with rare diseases we need to implement orphacodes, develop registries, and recognizing rare diseases as a public health priority. More than anything we need to recognize the immense knowledge that patients provide and include them on all levels.

**References**

1. Ferencz, B. It scares me how much of my son’s health depends on me. BMJ Opinion. 2018.

**Fig. 1 (abstract S4).**
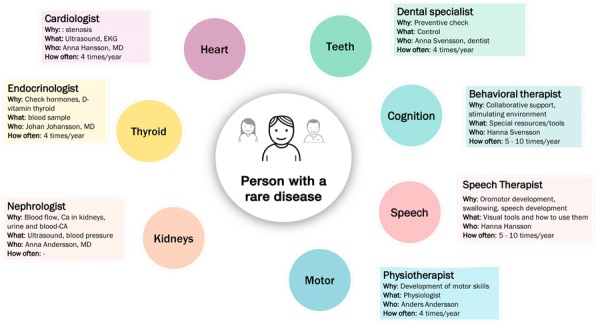
Example of a care map for a person with rare disease with points of contact and frequency

### S5 Failures to further developing orphan medicinal products after designation granted in Europe

#### Viviana Giannuzzi, Annalisa Landi, Adriana Ceci

##### Fondazione Gianni Benzi Onlus, Valenzano, 70010 Italy

###### **Correspondence:** Viviana Giannuzzi (vg@benzifoundation.org)

The research and development process in the field of rare diseases is characterised by many well-known difficulties. As a consequence, a large percentage of orphan medicines does not reach the market or the market authorisation is often granted on the basis of literature/retrospective data, i.e. outside a classic phase I-III scheme.

A recently published work identified orphan medicinal products designated in Europe under Regulation (European Commission) 141/2000 that failed to reach the market authorisation and investigated the stage of the development process at the time of its interruption, the reasons for and possible factors influencing failures [1]. EuOrphan and official sources were used and a literature search with a well-defined search strategy was performed. This study points out that about 28% failed the developmental process: 34 out of 788 designations failed the marketing authorisation process and 185 were abandoned during the development.

Most part of drugs getting to the marketing authorisation application reaches the phase III. In contrast, the majority of abandoned drugs apparently never started the development process, since no data on 48.1% of them were published and the 3.2% did not progress beyond the non-clinical stage.

With regards to the reasons for failures, the main ones were efficacy/safety issues, as generally happens for non-orphan medicines. However, also insufficient data, quality, regulatory and commercial issues raised in marketing authorisation failures; company inactivity/bankruptcy, change of company strategy and drug competition resulted further reasons for development failure. Unfortunately, no information was available for 23.2% of the analysed products. The paucity of publicly available information represents a problem still under debate, as it deals with the ‘transparency’ of results. In fact, Regulation (EC) 141/2000 dictates that the developmental stage of orphans has to be provided only to the European Agency on a confidential basis; furthermore, the publication of clinical trials, completion and discontinuation, in the EU registry has been made mandatory only with the new Regulation on clinical trials (EU) 536/2014.

Concluding, completing the R&D process still remains a challenging issue for orphan medicines.

Not only networking and collaboration, availability of public data and economic support/incentives, but also a stronger regulatory support and methodology to gain the reliable evidence supporting the marketing authorisation represent key ways to reduce the risk for failure of medicines for rare disease patients.

**References**

1. Giannuzzi V, Landi A, Bosone E, Giannuzzi F, Nicotri S, Torrent-Farnell J, Bonifazi F, Felisi M, Bonifazi D, Ceci A. Failures to further developing orphan medicinal products after designation granted in Europe: an analysis of marketing authorisation failures and abandoned drugs. BMJ Open. 2017 Sep 11;7(9):e017358. doi: 10.1136/bmjopen-2017-017358.

### S6 Global collaboration accelerates rare disease treatments

#### Tamara Howard^1^, Femida Gwadry-Sridhar^2^

##### ^1^Vice President of Patient Advocacy, Pulse Infoframe, Inc., Goring-on-Thames, Oxfordshire, RG8 9HD, UK; ^2^Chief Executive Officer, Pulse Infoframe, Inc., London, Ontario, Canada, N6G 4X8

###### **Correspondence:** Tamara Howard (thoward@pulseinfoframe.com)

Global ecosystems joining patients to clinicians and researchers and providing links for clinical trials, and new drug discovery, already exist. Some are run by charitable foundations, some by research organizations and some driven by patients, and all the examples mentioned here are powered by Pulse Infoframe Inc. (Pulse). Pulse focuses on rare diseases and cancers – which, given the identification of genetic subtypes – often fall into the categories of a rare disease. The company recognized many years ago that its disease focused ecosystems should be global and that the design should give something to all the participants.

The first such registry, the Global Melanoma Research Network, was set up in Canada in 2011 to join 12+ major research centres across Canada. More than 3,000 patients have taken part and their input provides relevant data to patient advocates for drug access and pharmaceutical companies to support the drug approval process. Researchers – and more than 48 scientists as well as 44 special physicians – have joined the network, resulting in changes to treatment for some sub-types of melanoma. All the data – more than 250 different pieces of data are collected for each patient – are organized into an ontology that facilitates acceptance by regulatory bodies such as the FDA and EMA.

The Orphan Disease Center at University of Pennsylvania has selected Pulse to power its global, rare disease registries, starting with CDKL5. Although very few in number (approximately 1,500 diagnosed patients around the world) ODC is spearheading global cooperation which has already sped up the pace of understanding of this disorder, with the goal of characterizing the spectrum of symptoms and heterogeneity of disease presentation and facilitating trial recruitment.

One very rare lymph node disease, Castleman Disease, now has a global registry than will soon span 5 continents. The drive to find a cure is strong as the creator of the registry is not only a physician and researcher, but also a patient with the disease [1]. This registry collects approximately 3,000 data points per patient and, as a result, this information has helped to characterize diagnostic tests for a disease which presents in multiple forms.

Collaboration cures disease – but every member of the disease focused ecosystem must have a voice and gain some benefit from taking part, particularly patients. Patients with rare diseases need a global registry to maximize the chances of making clinical progress for their conditions.

**References**

1. Thomas, K. His doctors were stumped. Then he took over. NYTimes. 2017. Retrieved from https://www.nytimes.com/2017/02/04/business/his-doctors-were-stumped-then-he-took-over.html

### S7 How to get research done on your rare disease

#### Daniel Lewi, Patricia Durão

##### The Cure & Action for Tay-Sachs (CATS) Foundation, London, SE12 0RW, UK

###### **Correspondence:** Daniel Lewi (dan@cats-foundation.org)

Driving research forward and initiating clinical trials into a rare disease can be undermined by challenges in patient recruitment and the difficulty in assembling large cohorts of affected individuals [1]. The International Rare Diseases Research Consortium (IRDiRC) is addressing this issue and recognising the scale of the “rare disease problem” has led to the realization that the time has arrived for global cooperation and collaboration among the many stakeholders active in rare diseases research [2].

At The Cure & Action for Tay-Sachs (CATS) Foundation we realised the key to advancing research to start any clinical trial into our rare diseases’ (the lysosomal storage disorders Tay-Sachs and Sandhoff which have an incidence rate of 1 in 320,000) was to identify those research teams focusing their work on them. We then identified that the next step was to start a collaboration with these teams with the aim to one day having a treatment.

Our role in this collaborative approach is an important one – we represent the patient by giving the disease a face and ensuring their best interests are at the forefront of all conversations. Due to the absence of resources from other sources, support from rare disease patient organisations can be crucial to the initiation and progress of biomedical research [3].

At The CATS Foundation we went a step further and set up the European Tay-Sachs and Sandhoff Charity Consortium (ETSCC) bringing together all the charities providing dedicated support and information on the diseases within Europe. Under the moto “One Group. One Goal. One Voice.” we have developed a clear and consistent strategy on how to help families provide care and how we can best fund research together as a consortium.

A collaborative approach to funding has enabled the charities to identify a variety of research projects that have the potential to treat Tay-Sachs and Sandhoff and the symptoms they cause. The success of this approach has seen us go from only one research project with a focus on a treatment for the diseases in 2011 to five potential treatment options being investigated in 2018. The ETSCC and its members are heavily involved in each of these projects.

We have a commitment to our rare disease community to provide children with these cruel diseases an opportunity to have a future, saving them from Tay-Sachs and Sandhoff disease. This drives our work forward and helps develop collaborations with research projects throughout Europe.

**Acknowledgements**

Thanks to Acción y Cura para Tay-Sachs (Spain), Hand in Hand gegen Tay-Sachs und Sandhoff (Germany) and Vaincre les Maladies Lysosomales (France) who are our co-member charities of The European Tay-Sachs & Sandhoff Charity Consortium (ETSCC).

**References**

1. Groft SC. Rare diseases research: expanding collaborative translational research opportunities. Chest. 2013; 144: 16–23.

2. Austin CP, Cutillo CM, Lau LPL, Jonker AH, Rath A, Julkowska D, Thomson D, Terry SF, de Montleau B, Ardigò D. Future of rare diseases research 2017–2027: an IRDiRC perspective. Clinical and Translational Science. 2018; 11: 21–27.

3. Pinto D, Martin, D, Chenhall R. The involvement of patient organisations in rare disease research: a mixed methods study in Australia. Orphanet Journal of Rare Diseases. 2016; 11: 2.

### S8 Digital transformation: everything is technically possible

#### David Martin Lindstrom

##### Device & Data Security at ElevenPaths, Telefónica, Madrid, Spain

Digital transformation is helping companies to be more efficient and create new and better services for its customers and users. The healthcare sector is also taking advantage of the new opportunities by incorporating technologies such as mobility, electronic imaging and electronic health records in company processes for diagnostics and treatments.

In this new context, the healthcare sector faces security challenges as a direct consequence of cyber-crime hitting the sector and where patients’ health and personal information is a lucrative goal for criminals.

### S9 Global Rare Equity: Argentina are we there yet?

#### Clarisa A Marchetti (Clarisa_marchetti@hotmail.com)

##### II Pharmacology Department School of Medicine Buenos Aires University, Paraguay 2155, Zip code C1121ABG CABA

The presentation is about to build collaborative strategies and the strengths and opportunities we offer to materialise them.

Key enablers

Argentina has 55 internationally recognized National Universities that receive students from all Latin America.

Argentina is a country with an open economy, receptive to international dialogue and accessible to the development of advanced therapies. Argentina has an innovative Science and Technology Ministry linked to the most important scientific institutions of the world.

Argentina has consolidated network of advocacy patients, validated by their protagonist and recognized of its representativeness.

A long tradition of excellence in scientific development allowed the formation of a highly qualified human resources

Argentina is a country with a prestigious career in medical sciences that has 3 Nobel prizes and we are looking for a fourth.

Opportunities in our health sphere:

National Law 26,689 of Rare Diseases, National Program of RD

National Registry of RD

Access to therapies

RedApta

2007; Creation of the Genetics Program

2009; RENAC: National Registry of Congenital Anomalies

2011; National Law of Rare Diseases: 26,689

2015; Regulation of the National Law

Current challenges; National Registry RD (SIISA), Consultative advice committee, List of RD

*Information sources:* Website of the Ministry of Health, Orphanet Argentina

*Training:* Annual RD Courses by Tele-health, RD in Primary Care in person

*Reference Centres:* Management leaders by province. Identification of RD expertise centres

*Interdisciplinary*: Advice and guidance to families, health teams. Joint work with NGOs.

*Measure Outcomes:* Preparation of reports.

Opportunities in our Science sphere:

Argentina understood the importance to accelerate the safe development of regenerative medicine.

*Legal & regulatory framework in Argentina*

*Professional Practice Medicine Act 1967*. Transplants Act 24.193. *Patient Rights Act 2009* Resolution 6677/2011 (Clinical Research)

*RED APTA*: Creation of a Coalition of patients in the Science Ministry. (Argentinean Patients Network for Advanced Therapies).

Argentina create the conditions to promote Regenerative Medicine providing a regulatory framework to research advanced therapies that facilitates the generation of safe and effective treatments for patients in harmony with international markets.

A collaborative case:

PAMPA *Consortium-Coalition for Precision Medicine with official support and endorsement (Ministries of Health and Science Tech and Innovation) framed in the National Precision Medicine Programme*

We will reach GLOBAL EQUITY with*:* Interdisciplinary, Collaborative team work, Health networks, Concerted Alliances.

Strengthening Health Systems Through Sustainable Interventions

Increase regional new-born screening practices

Education and Awareness

Harmonised data & digital technology

**References**

http://www.msal.gov.ar/congenitas/

http://www.celulasmadre.mincyt.gob.ar/institucional.php

http://fundacioninvestigar.com/

### S10 How to make exploitable research?

#### Lucia Monaco (lmonaco@telethon.it)

##### Research Impact and Strategic Analysis, Fondazione Telethon, Milan, Italy

In the rare diseases field, exploitation of research results bears the challenging goal of providing solutions to people living with diseases neglected by public research efforts and private development investments. Here, crucial factors are highlighted as key to accomplishing this goal, based on the experience of Fondazione Telethon in the development of a portfolio of gene therapies, starting from the rare genetic immunodeficiency ADA-SCID [1].

Creating the right environment. The strategic decision to invest in gene therapy by founding the SR-TIGET institute in 1995, in partnership with Ospedale San Raffaele in Milan, created an ideal research and clinical environment and allowed the recruitment and support of leading scientists and clinicians.

Promoting excellent research to elucidate the disease mechanism, to study the disease natural history and to reach proof of principle of the therapeutic approach in reliable animal models. Continued financial support relied on rigorous review of the proposed activities and monitoring of results.

Developing the appropriate gene therapy platform and investing in the production of the medicinal product according to good manufacturing procedures. This step enabled a clinical trial complying with regulatory requirements.

Acquiring orphan drug designation (ODD) for the ADA-SCID gene therapy. ODD was obtained from both the European Medicines Agency and the American Food and Drug Administration, as it was deemed crucial to secure market exclusivity for prospective development partners.

Performing a phase I/II clinical trial according to good clinical practices. The first ADA-SCID patient was treated by the SR-TIGET team in 2002; in 2009 the therapy was proven safe and effective for all 12 children treated [2].

Partnering with pharma to reach marketing authorization. The alliance with GlaxoSmithKline in 2010 made the completion of the development process possible, thanks to the company’s competences and resources, which complemented those by SR-TIGET, and covered other gene therapies being developed within the institute. The therapy for ADA-SCID gained marketing authorization in Europe as Strimvelis® in May 2016 [3]. The alliance set an example for subsequent partnerships with other companies, safeguarded researchers’ independence, kept intellectual property rights with Fondazione Telethon and Ospedale San Raffaele and included binding conditions for therapy development by the company.

In conclusion, the ADA-SCID case illustrates some key enabling factors for competitive/transformative research, which span from the promotion of excellent fundamental and pre-clinical research, to the identification and support of projects with translational potential, to the engagement in the fundamental steps for effective translational research.

**References**

1. Monaco L, Faccio L. Patient-driven search for rare disease therapies: the Fondazione Telethon success story and the strategy leading to Strimvelis. EMBO Mol Med. 2017; 9:289-292.

2. Aiuti A, Cattaneo F, Galimberti S, Benninghoff U, Cassani B, Callegaro L, Scaramuzza S, Andolfi G, Mirolo M, Brigida I, Tabucchi A, Carlucci F, Eibl M, Aker M, Slavin S, Al-Mousa H, Al Ghonaium A, Ferster A, Duppenthaler A, Notarangelo L, Wintergerst U, Buckley RH, Bregni M, Marktel S, Valsecchi MG, Rossi P, Ciceri F, Miniero R, Bordignon C, Roncarolo MG. Gene therapy for immunodeficiency due to adenosine deaminase deficiency. N Engl J Med. 2009; 360:447-58.

3. Aiuti A, Roncarolo MG, Naldini L. Gene therapy for ADA-SCID, the first marketing approval of an ex vivo gene therapy in Europe: paving the road for the next generation of advanced therapy medicinal products. EMBO Mol Med. 2017; 9:737-740.

### S11 Cross Border Health: ERN-EuroBloodNet’s overview for reaching an equal access to care for rare haematological diseases patients across European Union’ Members States

#### María del Mar Mañú Pereira^1^, Victoria Gutiérrez Valle^2^, Mariangela Pellegrini^2^

##### ^1^University Hospital Vall d'Hebron - Vall d'Hebron Research Institute, 08035, Barcelona, Spain; ^2^AP-HP Hôpital Saint Louis, Paris, 75010, France

###### **Correspondence:** Mariangela Pellegrini

**Background**

Due to the scarcity of patients and knowledge, rare diseases, affecting less than 1 in 2000 individuals, are the area in public health in which joint efforts among European Member States is most crucial. ERN-EuroBloodNet, the European Reference Network on Rare Haematological Diseases (RHD), results from a joint effort of the European Network on Rare and Congenital Anaemias (ENERCA), the European Haematology Association (EHA), and European haematology patient organisations represented in both the EURORDIS European Patient Advocacy Groups (ePAGS) and the EHA Patient Organisations Workgroup. ERN-EuroBloodNet gathers 66 highly skilled and multidisciplinary healthcare teams in 15 Member States. Aimed at facing the challenges of RHD, it gathers also advanced specialized medical equipment and infrastructures which will facilitate concentration of resources for the design, validation and implementation of high-quality and cost-effective services.

**Methods**

Methods and tasks aiming to achieve ERN-EuroBloodNet specific objectives have been split into five categories of Transversal Field of action (TFA): 1) Cross Border Health to establish a referral system for patients and samples in order to ensure the same level of access to healthcare across Europe. 2) Best practices to compile, create, and assess their implementation, alongside disseminating guidelines in RHD 3) Continuing medical education to spread cutting-edge knowledge in the field of RHDs. 4) Tele-medicine to facilitate inter-professional consultation by sharing of expertise and safety exchange of clinical information. 5) Clinical Trials and Research to foster European cooperation for epidemiological surveillance, development of high specialized procedures for diagnosis, innovative treatments and research.

**Results**

TFA on Cross Border Health (CBH) is strictly connected with the Directive 2011/24/EU adopted by the European Union regarding the application of patients’ rights in cross-border healthcare and including article 12 regarding the establishment of ERNs based on the recognition of Centres of expertise at national level [1]. In order to obtain a solid analysis, some tests have been launched, i.e. questionnaire on NGS-highly specialized diagnostic and surveys on CBH Barrow Marrow Transplantation for non-malignant disorders.

Conclusions

After first analysis, conclusions reached agree with the position that to give equal and highly qualified access to care at a European level means to maximizing the cost-effective impact on RHDs.

**Keywords**

European Reference Networks, cross border health directive, rare haematological diseases’ economic impact, and equal access to healthcare services.

**Acknowledgements**

This abstract is presented on behalf of the coordination group of the ERN-EuroBloodNet. It is led by Prof. Pierre Fenaux from Ht St Louis in Paris and Prof Béatrice Gulbis from Ht ERASME-ULB- Cliniques Universitaires de Bruxelles in Brussels. ERN-EuroBloodNet Scientific Director, Dr Maria del Mar Mañú Pereira, IT and dissemination manager, Ms Victoria Gutierrez Valle, both from University Hospital Vall d'Hebron - Vall d'Hebron Research Institute in Barcelona and ERN manager, Dr Mariangela Pellegrini, from Ht St Louis complete the ERN-EuroBloodNet coordination team.

**References**

1. Cf. Directive 2011/24/EU of the European Parliament and of the Council of 9 March 2011 on the application of patients’ rights in cross-border healthcare (OJ L 88, 4.4.2011, p. 45–65)

### S12 The digital patient – the research behind collaborative care and analytics

#### Ivo R. M. Martins

##### Atos Research and Innovation – Health sector, Atos, Madrid, 28037, Spain

Vienna, 11th of May of 2018, EURORDIS - 9th European Conference on Rare Diseases & Orphan Products. The author participated on the debate of “Theme 3 Digital Patient - Everything is Technically Possible” presenting examples of research projects. The digital patient creation and evolution was the main topic of the theme, namely, the obstacles to create digital resources and interactions in the health field, existing technologies and how they can modernize and offer innovative solutions to improve the patients’ daily lives and develop the medical research. The examples presented were related with the collaborative care and analytics matters. The collaborative care is linked with telemedicine being integrated increasingly in clinical routine and the analytics proposes to give policy makers and clinicians the comprehension needed to take informed decisions.

The HarmonicSS project is an example of collaborative care. It aims on bringing together regional, national and international cohorts of patients suffering of Primary Sjögren’s Syndrome (pSS), with the inclusion of patients in clinical trials and taking into account the ethical, legal and privacy matters of data sharing from different countries, building a harmonized cohort. This will help the detection of clinical and health policy pSS unmet needs with the use of open services for big data mining, governance and visual analytics. The users of the platform are researchers, clinicians, health policy makers and pharmaceutical companies [1].

The next example is pocket mHealth, an Atos start-up initiative that is composed by a smartphone and a desktop application, installed at the medical centres and hospitals, providing universal and validated clinical information access at any time, bringing and managing the Electronic Health Record (EHR) in the smartphone validated by the medical professionals. Based on eHealth standards, allows the integration of clinical data coming from heterogeneous Hospital Information Systems (HIS), improving diagnosis, suppressing extra paper or DVD reports, avoiding duplications and unnecessary tests [2].

Finally, CrowdHealth project was presented as an example of analytics. The purpose is to deliver a platform that provides decision support to public health authorities for policy creation, from the exploitation of collective knowledge and its permutation with situational awareness artefacts. With the use of casual and risk stratification mechanisms and merging with simulation tools, it will simplify policies evaluation and optimization over visualizations of simulations and outcomes of evidence based analysis of prevention strategies [3].

**References**

1. Harmonicss consortium. Harmonicss document of work - Harmonization and integrative analysis of regional, national and international Cohorts on primary Sjögren’s Syndrome (pSS) towards improved stratification, treatment and health policy making, document of work.; 2016. p.118-130.

2. Cavero, C. (2016). Your Health always with you [online] Avaliable at: http://www.pocketmhealth.com/en [03/05/2018]

3. Crowdhealth consortium. Crowdhealth document of work - Collective wisdom driving public health policies.; 2016. p.69-92.

### S13 Transformational diagnostic pathways: conversation between patients and clinicians/researchers: A family with no diagnosis

#### Alessandra Renieri^1,2^, Elisa Frullanti^1^

##### ^1^Medical Genetics, University of Siena, Siena, Italy; ^2^Genetica Medica, Azienda Ospedaliera Universitaria Senese, Siena, Italy

I was talking with Louise James, mother of an undiagnosed children, suggesting strategies for diagnosing of undiagnosed (exome-negative) diseases. Exome sequencing is increasingly used in the diagnosis of disorders of unknown genetic aetiology and exome-negative patients are an emerging category of undiagnosed patients. There are a number of reasons why they are exome-negative and in Vienna at ECRD 2018, I explained to patient organizations what can be done in these cases.

What is/should be the follow up be for exome-negative patients (Fig. 1)?

i) go back to patient phenotyping and think about candidate genes (the whole exome could be “holed” not providing coverage over all exonic regions);
ii) re-analyse (yearly) the stored exome data updating according to new scientific findings;
iii) do RNA/functional analysis of candidate gene by connecting with experts;
iv) do genome sequencing analysis if all the previous strategies were negative.

Consent for publication

The authors declare that they have received written informed consent to publish from the participants.

**Fig. 1 (abstract S13).**
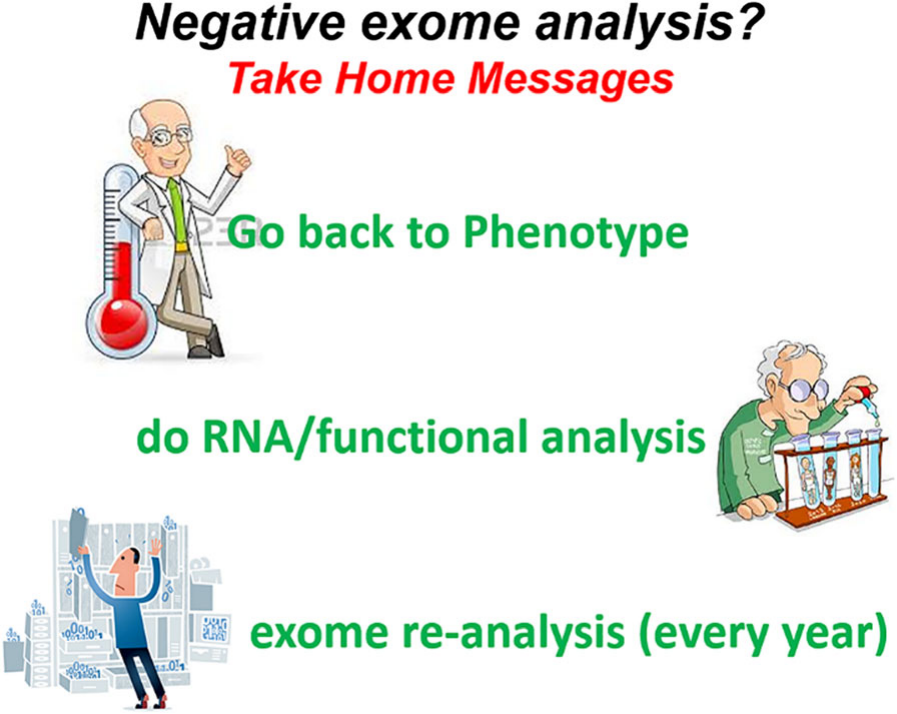
Take Home Messages

### S14 Understanding and using patient experiences as evidence in healthcare priority setting

#### Leah Rand^3^, Michael Dunn^2^, Ingrid Slade^2^, Sheela Upadhyaya^3^, Mark Sheehan^2^

##### ^1^National Academy of Sciences, Washington D.C., USA; ^2^Ethox Centre, University of Oxford, Oxford, UK; ^3^NICE, Manchester, UK

###### **Correspondence:** Sheela Upadhyaya

In many countries, committees make priority-setting decisions in order to control healthcare costs. Each such committee takes into account relevant criteria for its decision, including clinical effectiveness, cost-effectiveness, and need. Whether these criteria give the committee a reason to fund coverage of a treatment depends on the evidence that supports them. Usually that evidence is drawn from scientific, epidemiologic, and economic studies. However, criteria like need—understanding the nature of the condition and its effects on patients and caregivers—can best be supported by evidence generated from patients’ experiences. This is especially the case for rare diseases where small populations may make robust clinical evidence scarce.

Patient experience has an important role to play informing healthcare decision making. Patient experience as evidence is distinct from patient involvement and the reasons that justify it. Rather than rest on democratic grounds, like involvement, patient experience is required for a decision-making process that considers all relevant evidence. Priority-setting processes should be set up so that a fair decision is made. In order for the decision to be fair, the decision makers should consider evidence and reasons relevant to the decision. Such a decision would be questionable if it ignored clinical trials evidence, and similarly, it is unfair to ignore the body of relevant evidence from patient experience. Considering all relevant evidence and reasons gives each person an equal opportunity at having their treatment funded. Patient experience not only gives context to the clinical evidence, it also directly informs our understanding of the nature of the condition and its effects on patients and carers, including what their needs are, how those needs could be met, and the burden of illness on patients and carers. Such evidence also serves to contextualise reported effects of the treatment.

In order for patient experience to inform decisions, it must generate high quality and methodologically rigorous evidence. In response to concerns about relying on anecdotes or individual narrative, quantitative and qualitative methods can be adopted from the social sciences in order to assure that evidence is systematic, adherent to quality standards, and valid. Interviews can refine research questions and scope while surveys provide generalisable information. Patient, like clinical, evidence should be reviewed in a transparent evidence review process. With robust methods and research oversight, patient experience can generate valid evidence important to the priority-setting process.

